# Graphene Quantum Dots: Novel Properties and Their Applications for Energy Storage Devices

**DOI:** 10.3390/nano12213814

**Published:** 2022-10-28

**Authors:** Sajid Ali Ansari

**Affiliations:** Department of Physics, College of Science, King Faisal University, P.O. Box 400, Hofuf 31982, Saudi Arabia; sansari@kfu.edu.sa; Tel.: +966-1-3589-9598

**Keywords:** graphene quantum dots, energy storage, batteries, supercapacitors, electrode material

## Abstract

Batteries and supercapacitors are the next-generation alternative energy resources that can fulfil the requirement of energy demand worldwide. In regard to the development of efficient energy storage devices, various materials have been tested as electrode materials. Graphene quantum dots (GQDs), a new class of carbon-based nanomaterial, have driven a great research interest due to their unique fundamental properties. High conductivity, abundant specific surface area, and sufficient solubility, in combination with quantum confinement and edge effect, have made them appropriate for a broad range of applications such as optical, catalysis, energy storage and conversion. This review article will present the latest research on the utilization of GQDs and their composites to modify the electrodes used in energy storage devices. Several major challenges have been discussed and, finally, future perspectives have been provided for the better implementation of GQDs in the energy storage research.

## 1. Introduction

Due to the ever-increasing environmental pollution and energy crisis, the development of sustainable and clean energy resources has become urgent in the 21st century [1,2]. This demands the fabrication of low-cost even high-performance energy storage and energy conversion devices. In this regard, the fabrications of efficient energy resources, especially electrochemical energy storage devices, have attracted the research community in recent years [1,2,3,4,5]. The performance of such energy storage devices strongly relies on the nature of the electrode materials applied. To date, many efforts have been made to imply different types of materials and find out their suitability in developing real-life tools for energy storage and conversion [5,6,7,8,9,10,11,12]. Among them, two-dimensional graphene, an important allotrope of carbon, is widely used for energy storage applications [12,13,14]. The sp^2^-hybridized conjugated structure, along with interesting mechanical, electrical and thermal properties, make them a distinctive class of material with versatile applications. Additionally, they offer high surface area, sufficient chemical stability, and low-cost fabrication, which are also desirable for constructing an efficient device [15,16,17,18,19]. Importantly, graphene sheets can be cut down into a few nanometers, especially less than 10 nm, giving rise to interesting physicochemical properties. Typically, the graphene sheets below 10 nm are called graphene quantum dots (GQDs). Unifications of the interesting properties of graphene and quantum dots lead to evolving unique physicochemical properties in GQDs that confer their promises for successful applications in optoelectronics, energy storage, and conversion applications [20,21,22,23,24].

**Figure 1:**
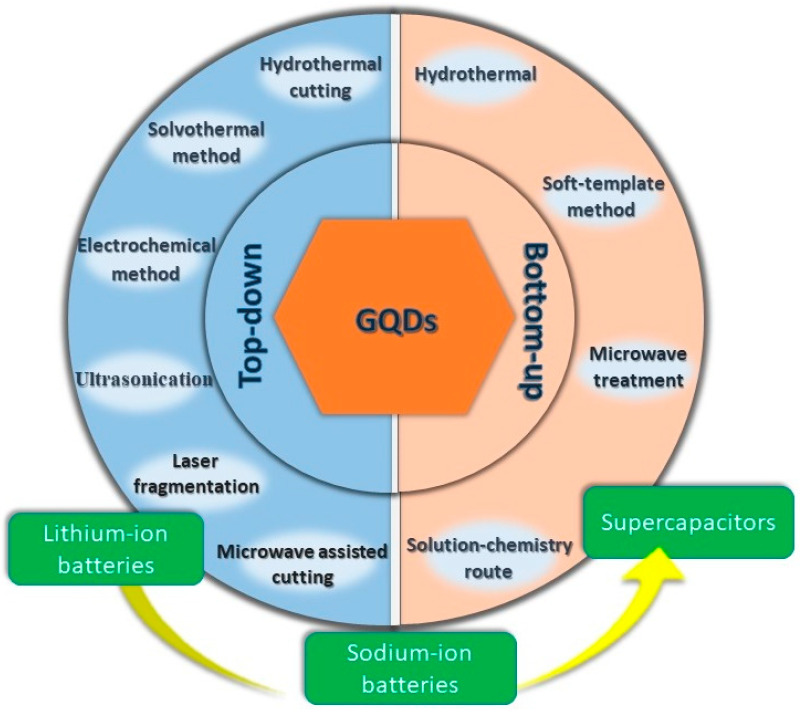
Fabrication and diverse application of the GQDs.

Although several efforts have been made up to date, the research and development on the synthesis of GQDs is still at an early stage [25,26]. It is well known that GQDs are very good optical materials [27,28]. Besides, with an aim of expanding their utility in energy-related applications, a tremendous focus on developing GQD-based composite materials for electrochemical energy storage applications can be seen in recent years (Figure 1) [29,30,31,32]. In this regard, a comprehensive review article demonstrating the recent advances in the composition and utilization of GQDs and their composites for efficient batteries and supercapacitors electrodes is important and necessary. Herein, this review article highlights the recent discoveries of novel synthesis protocols, underlying synthesis mechanisms, and their effects on the GQDs’ structural and surface properties. Importantly, the presence of the defect states, doped heteroatoms, surface groups, and conductivity of the material can have a significant influence on the electrochemical energy storage phenomena which have been barely discussed in detail by other review articles. This review article systematically elaborates on the effect of the aforementioned parameters on the charge storage performance of GQD-based electrodes. In addition, the interaction of GQDs with other materials, and their crucial role in enhancing the performance of the produced nanocomposite electrodes, are revealed in this article. These outlooks are expected to provide a deeper understanding of the successful applications of GQD-based electrode materials for developing cost-effective and environmentally safe future energy storage systems.

## 2. Synthesis of GQDs

The synthesis process of GQDs can be broadly divided into two categories: top-down and bottom-up approaches. A top-down approach corresponds to the decomposition of bulk, readily available graphene-based materials. Smaller particles are formed under harsh conditions involving high temperatures. However, such methods lack distinct control over particle size distribution and the morphology of the as-produced particles. A bottom-up approach, on the other hand, involves the synthesis of QDs with the help of chemical reactions from the molecular species. Such a method allows for achieving excellent control over the particle size and fundamental properties of the material. In this section, we shall discuss efficient synthesis techniques that have been reported very recently for GQD preparation.

### 2.1. Top-Down Approach

Typically, a top-down approach for the synthesis of GQDs involves the cleavage of larger carbonaceous structures such as graphite, carbon fiber, carbon black, coal, and graphene oxide (GO). So far, various top-down strategies have been applied to successfully synthesize GQDs, and in this section we shall discuss some of the useful strategies.

#### 2.1.1. Hydrothermal Cutting

**Figure 2:**
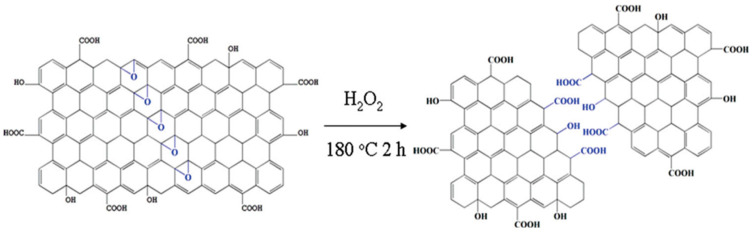
Rapid hydrothermal cutting of GO into GQDs. Superoxide anions assisted in cutting the C–C bond of vicinal diol. Reprinted with permission from Ref. [37].

A hydrothermal process involves cutting off larger carbonaceous structures in presence of an aqueous alkaline solution. Such a technique was first employed by Pan et al. to prepare water soluble luminescent GQDs [33]. In this process, the synthesis of GQDs involves thermal deoxidization of GO sheets to graphene sheets (GSs), followed by acid oxidation of GSs and hydrothermal treatment. Overnight dialysis of the as-produced colloidal solution yielded strongly fluorescent (blue emission) GQDs. Pan et al. proposed that acid oxidation generated epoxy groups in a line by rupturing C–C bonds throughout the carbon lattice. Once the epoxy chains were formed, they were further oxidized to more stable carbonyl groups under room temperature conditions. Such oxidation of GSs was confirmed by FTIR spectroscopy indicating a strong carbonyl signal. Subsequently, the hydrothermal deoxidization step removed the bridging carbonyl oxygen atoms to form GQDs with 5–13 nm diameter. Hydrothermal treatment was done at 200 °C temperature and pH 8 for 10 h. The cutting efficiency was ~5 wt% of the precursors. Furthermore, the same group showed that well crystallized structures can be produced at a higher thermal deoxidization temperature [34]. Cutting of GO sheets at a relatively higher temperature not only resulted in the ordered structures but, also, the wavelength of emission was red-shifted (blue emission), as compared to the previous result, and smaller size particles (diameter of 1.5–5 nm) were formed. Sun et al. reported direct oxidation and etching of graphite powder for the scalable and efficient synthesis of GQDs [35]. In this study, they prepared GQDs by following the method of Hummers and Offeman with several modifications. They increased the NaNO_3_/graphite mass ratio from 2:1 (for the Hummers method) to 43:1, and the reaction temperature was increased from 35 to 120 °C. In a typical synthesis process, 1.0 g of graphite and 43.0 g of NaNO_3_ were added to 100 mL concentrated sulfuric acid, and the resultant mixture was cooled to 0 °C. Afterwards, 3.0 g of KMnO_4_ was added to form graphite oxide. Cutting of graphite oxide at 120 °C resulted in the formation of tiny dots, and the yield was measured to be as high as 63 ± 7 wt%. Although the final yield of the product was improved significantly by following this procedure, the QY was only 3%. Lin’s group used potassium superoxide (KO_2_) as a “scissor” to cut the GO sheet under hydrothermal conditions. The conversion rate was ~35 wt% from GO with 8.9% QY. Experimental results suggested that epoxy groups tend to appear along a line throughout the carbon lattice. Subsequently, cooperative alignment of the epoxy groups produces a strong tension as a result of which underlying C–C bonds tend to break [36]. Hydrogen peroxide (H_2_O_2_) can act as an efficient “scissor” for cutting the GO sheet during hydrothermal synthesis. It is important to mention that such free radical initiators are relatively less hazardous as compared to strong acids and abundantly produced oxygenated groups that induce exciting photophysical properties and good solubility. It was found that H_2_O_2_ treatment could reduce the reaction time to only 2 h (Figure 2), and as-prepared GQDs (size—1.5–5.5 nm) showed bright excitation independent emission with up-conversion properties [37]. On the other hand, hydrothermal reaction time can be further reduced to a few seconds by using the continuous hydrothermal flow synthesis (CHFS) process in combination with supercritical water and *p*-tetrasulfonic acid calix [4] arene [38].

#### 2.1.2. Solvothermal Method

**Figure 3:**
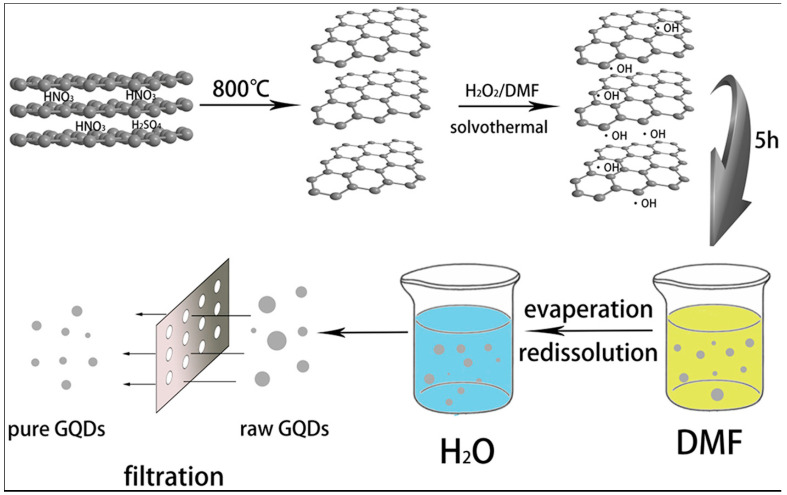
Fabrication of graphene quantum dots by hydrogen peroxide. Reprinted with permission from Ref. [39].

Typically, a solvothermal process involves the use of an organic solvent and treatment of the reaction mixture in a Teflon-lined autoclave at a higher temperature (Figure 3). For example, Yang’s group dispersed GO in N,N-Dimethylformamide (DMF), and the solution was subjected to ultrasonication for 30 min. After that, the GO/DMF solution was transferred to a Teflon-lined autoclave for heating at 200 °C for 8 h. After the synthesis, the product was purified via column chromatography. The as-produced GQDs were highly soluble in several polar solvents under different pH levels and showed a maximum of 12.2% QY [39].

However, Fang et al. treated the GO/DMF solution for 5 h at 200 °C and were able to obtain strong green emissions with 23.1% QY [40]. Also, Tian et al. reported good QY for GQDs during the solvothermal synthesis [41]. In this report, they used expanded graphite as the precursor. Firstly, expanded graphite was mixed with DMF, ultrasonicated for 5 min, and then H_2_O_2_ was added to the solution. Heating up to 170 °C for 5 h resulted in the formation of GQDs with strong blue emissions (15% QY). For purification of the product, the solution was passed through a 100 nm filter membrane so that unreacted expanded graphite was removed from the solution. The process was quite simple because no complicated equipment was needed for the synthesis, and the purification stage was easier as well. A recent study shows that potassium monopersulfate, also called oxone, can be used as an efficient oxidant during solvothermal synthesis [42]. Four different natural resources such as charcoal, carbon fibers, graphite, and multi-walled carbon nanotubes were chosen. It was reported that oxone can successfully synthesize the GQDs from all these four different kinds of carbon sources. During the synthesis, sulfate radicals (SO•^−^) or hydroxyl radicals (OH•) were generated by using the oxone oxidizing agent. The mechanism was associated with radical oxidation, followed by solvothermal reduction to cut the C–C bonds. Radical-initiated oxidation followed by cleavage and, finally, the reduction of the larger size carbon materials resulted in the formation of very small-sized GQDs. There are several advantages in such a method. This is an acid-free technique eliminating the step of strong acid neutralization. Furthermore, the purification stage is simple and eco-friendly, offering a high yield suitable for large-scale production.

#### 2.1.3. Electrochemical Method

**Figure 4:**
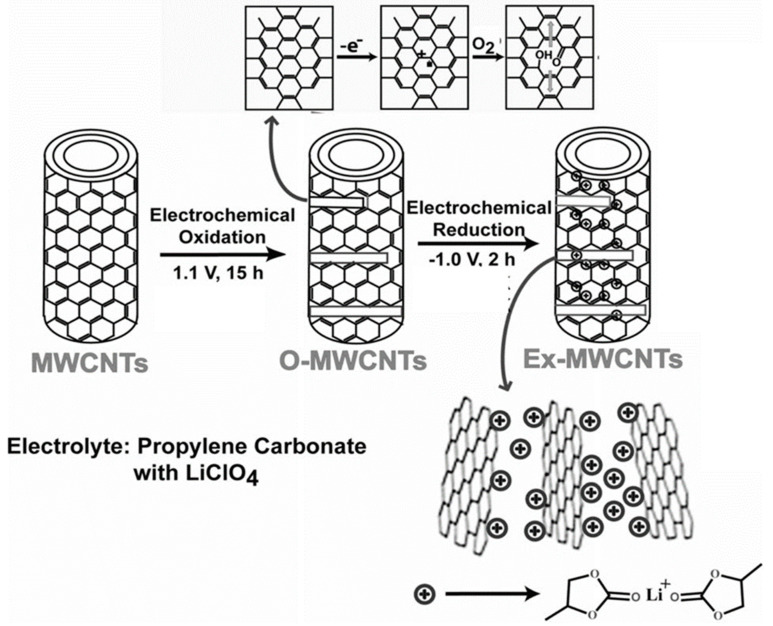
Electrochemical cutting of MWCNTs to prepare GQDs. The process is associated with electrochemical oxidation in step 1 followed by an electrochemical reduction in step 2. Reprinted with permission from Ref. [43].

Several research groups have used electrochemical techniques for cutting larger carbonaceous structures. Such a method, with potentiostatic oxidation followed by reduction, offers excellent control over GQDs size and shape. Pillai’s group reported a two-step method for the transformation of multi-walled carbon nanotubes (MWCNTs) into GQDs [43]. Figure 4 represents an electrochemical transformation process. Here, at first, an anodic potential of 1 V (for 15 h) vs. Pt quasi reference electrode (QRE) was applied to a MWCNT-coated working electrode, resulting in the formation of oxidized MWCNTs. During the process, propylene carbonate with LiClO_4_ was used as an electrolyte. Next, the application of a −1 V potential (for 2 h) vs. Pt QRE induced intercalation of the complexes of Li^+^/propylene carbonate. As a result of such intercalation, exfoliation of oxidized MWCNTs took place and, finally, GQDs were formed at a 90 °C temperature. Microscopic results suggested that GQDs with 3 ± 0.3, 5 ± 0.3, and 8.2 ± 0.3 nm size distribution were formed after the electrochemical exfoliation and heat treatment at 90 °C/15 h, 90 °C/11 h, and 90 °C/7 h, respectively. Interestingly, exfoliation with propylene carbonate by using LiClO_4_, and heating at a relatively lower temperature, such as the 30 °C temperature, resulted in the formation of larger size carbonaceous nanoparticles (size distribution 23 ± 2 nm). Thus, the applied temperature during the electrochemical synthesis plays a crucial role in controlling the particle size as well.

Red emissive GQDs were prepared by electrolysis of graphite in the presence of 0.01 M K_2_S_2_O_8_ aqueous solution. Here, electrolysis was done at pH 7 + 5.0 V. Also, the current intensity was varied between 80 and 200 mA cm^−2^ for the GQDs’ preparation. Experimental results suggested that OH• and O• were produced due to the anodic oxidation of water. They served as electrochemical “scissors” to cut the graphite into graphene sheets [44]. Li et al. used sodium phytate (0.1 mol/L), and applied a +5 V scanning voltage for 12 h, to prepare GQDs with phosphorus (P) doping. They used a high-purity graphite rod as the working electrode and a platinum electrode as the counter electrode. Utilization of such an electrochemical technique caused a sufficient amount of phosphorus (P) doping inside the GQDs’ structure, and the as-prepared P-GQDs showed excellent free radical scavenging ability and anti-erosion performances [45]. Recently, a three-electrode system has been utilized for the successful preparation of GQDs at room temperature. 20 µL of GO (1mg/mL) was drop casted over a Glassy carbon electrode (GCE) to prepare a working electrode. A Pt film was used as the counter electrode and a Pt wire was used as the QRE. A potential of +1.05 V was applied in propylene carbonate-LiClO_4_ (3 mM) electrolyte for the oxidation of GO. After the oxidation step, the potential was set at −1.05 V for 3 h for the electrochemical reduction. Finally, the GCE electrodes were sonicated to collect the as-produced GQDs. Such a process gives an excellent opportunity to control the size of GQDs by tuning different parameters such as redox time, supporting electrolyte concentration, and applied potential [46].

#### 2.1.4. Ultra-Sonication

In this process, ultrasound energy is applied to agitate larger carbonaceous structures during the synthesis. For example, ultrasonic treatment (45 W, 59 kHz) of acetylene black dispersed in 30 mL of N-methyl-2-pyrrolidone (NMP) solvent for 1 h resulted in the formation of GQDs. For the purification stage, centrifugation at 10,000 rpm was done, and excess NMP solvent was removed by applying vacuum evaporation at 100 °C [47]. In another work, graphene was oxidized at room temperature in the presence of concentrated HNO_3_ and H_2_SO_4_ for 12 h. After that, the reaction mixture was subjected to ultrasonic treatment (300 W, 80 kHz) for 12 h and calcined at 350 °C to remove excess concentrated acids. GQDs with 3 to 5 nm diameters were recovered by using this method after the filtration and dialysis of the final product solution [48]. Zhang et al. showed that anthracite coal can also be used as a precursor for the ultrasonic synthesis of GQDs. They took 200 mg of anthracite coal in 50 mL of DMF, and the suspension was treated with a 21 kHz ultrasonic wave for 2 h. Subsequently, the suspension was passed through a 0.22 μm membrane and dialyzed for 3 days to remove excess solvent. Here, particles with 3.2 ± 1.0 nm diameter were successfully recovered after the purification stage which showed good fluorescence as well [49].

#### 2.1.5. Laser Fragmentation

**Figure 5:**
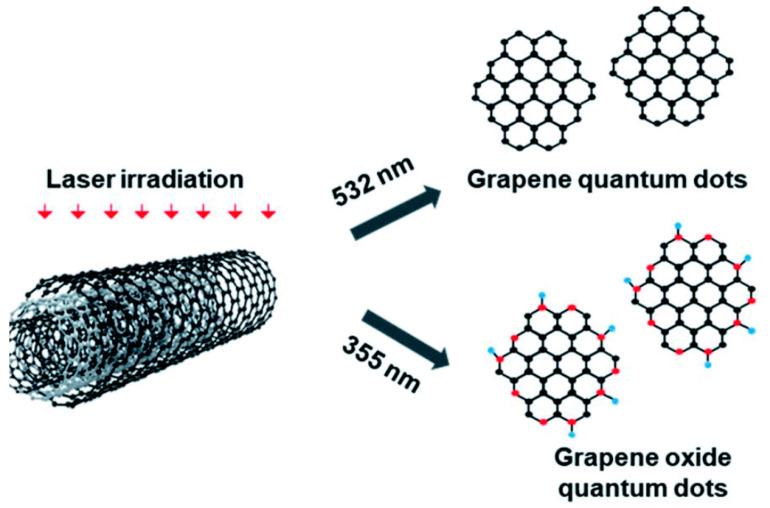
A schematic illustration for the generation of GQDs from PLAL process. Reprinted with permission from Ref. [50].

**Figure 6:**
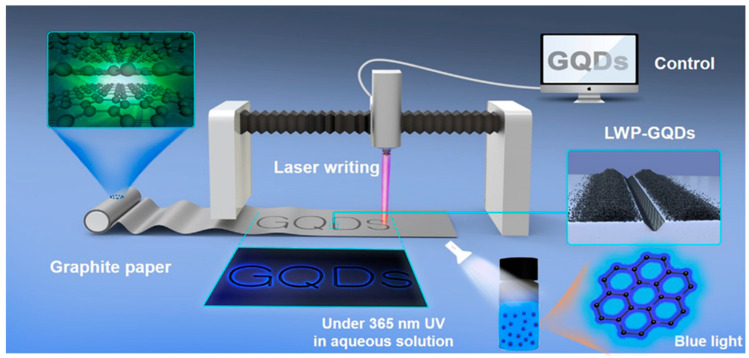
A schematic illustration for the generation of laser writing produced GQDs. Reprinted with permission from Ref. [52].

Laser-assisted fragmentation of the larger carbonaceous structure is one of the most popular top-down approaches to synthesize GQDs. Such a process refers to breaking down a certain portion of a material by using a laser beam of a particular wavelength. MWCNTs (Figure 5) [50], graphene sheets [51], graphite paper [52], carbon nano-onions [53], and coal [54] are the commonly used precursors for such a synthesis technique. Kang and co-workers chose MWCNTs in high-purity ethanol and employed a ND:YAG laser beam to the suspension [50]. Here, MWCNTs were irradiated with 355 nm and 532 nm pulsed laser sources (ablation energy 50 mJ and 10 Hz repetition rate). GQDs with <5 nm diameter were formed after 10 min of ablation by using a 532 nm laser source. However, when higher photon energy was applied (λ = 355 nm), GOQDs were formed. A laser beam with high photon energy photo-thermally decomposed the solvent forming the cavitation bubbles which consisted of carbon- and/or oxygen-based small molecules over the MWCNT surface. Such a result suggested that GQDs could be easily functionalized with oxygeneous species simply by changing the ablation wavelength. In another study, a 1064 nm laser beam was utilized to irradiate the graphene sheet [51]. Laser irradiation for 40 min resulted in the formation of a light-yellow color solution. After the centrifugation and pH adjustment, the obtained transparent solution was subjected to hydrothermal treatment at 150 °C for 12 h. Small particles with uniform size distribution were formed after the completion of reaction. Figure 6 corresponds to the schematic representation for GQDs’ production by using a laser writing instrument [52]. In this process, by changing the output power of the incoming laser radiation, GQDs were successfully generated from graphite paper.

Here, the formation of GQDs was attributed to the power of laser irradiation, which caused photothermal vaporization to generate gaseous graphene. Subsequently, graphene vapour was recrystallized to generate ultrafine particles of GQD. Dispersion of the as-produced GQDs showed blue fluorescence after excitation with a 365 nm light source. Notably, the yield of the final product could be easily controlled by varying the output laser power and laser scan speed. This novel method was highly efficient, ultra-fast, and suitable for large-scale production.

#### 2.1.6. Microwave Assisted Cutting

Recently, the microwave technique has become a common method of nanomaterial synthesis because of several advantages, such as (1) Short reaction time, (2) Homogeneous heating of the reaction mixture, and (3) good yield and high purity of the final product. Such a technique has also been employed for the top-down GQDs synthesis. Li et al. reported a one-pot microwave-assisted technique to produce greenish-yellow emission GQDs [55]. Here, GO nanosheets were treated in the presence of concentrated HNO_3_ and H_2_SO_4_ for 3 h. Further reduction of GQDs in the presence of NaBH_4_ changed the luminescence to intense blue. The maximum QYs were measured to be 11.7% and 22.9% for greenish-yellow GQDs and blue GQDs, respectively. Interestingly, NaBH_4_ reduction induced no perceptible changes to the dimension and height of GQDs. Thus, shifting in the emission peak was attributed to structural changes, rather than dimension variations. Sun et al. took a different approach to synthesis. They combined the microwave technique with hydrothermal treatment and took fluorinated graphene oxide (FGO) as the starting material [56]. In this technique, a solution of FGO (concentrated HNO_3_ and H_2_SO_4_) was heated under microwave irradiation at 650 W for 6 h. Following synthesis, the reaction mixture was cooled to room temperature, ultrasonicated for a few minutes, and then the medium pH was adjusted to 6. After the filtration, followed by dialysis of the product solution, fluorinated GQDs were recovered. Next, the precipitate obtained during the purification stage was collected and redispersed in water. pH was adjusted to 8. The aqueous suspension was then transferred to a Teflon-lined autoclave and was heated at 200 °C for 10 h to form blue fluorescent GQDs. Ultrahigh photostability, very good pH stability, and improved product yield (~10%) were achieved by using such a technique.

Furthermore, the utilization of chemical exfoliation [57,58] and lithography technique [59,60] can be found for scissoring of the graphene sheets via top-down approach.

### 2.2. Bottom-Up Approach

**Figure 7:**
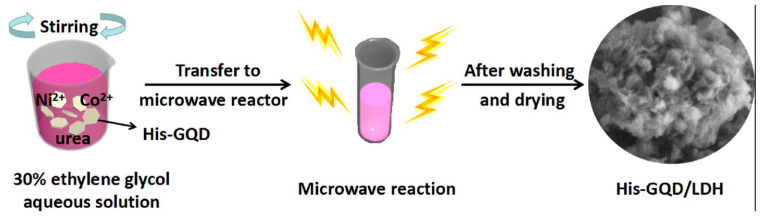
Illustration of the microwave synthesis of graphene quantum dots/Ni-Co LDH. Reprinted with permission from Ref. [65].

In a bottom-up technique, small organic molecules are used as the starting materials. Here, chemical reactions take place among the precursor molecules to form a graphitic core inside the QD system [61,62,63,64,65]. In this section, we shall discuss the bottom-up approach to the synthesis of GQDs. For the bottom-up synthesis, various research groups have shown that the treatment of small organic molecules in the presence of microwave radiation or a hydrothermal environment result in the formation of GQDs with good production yield and valuable photophysical properties [65,66,67,68,69,70,71,72,73,74,75,76,77,78,79,80]. Blue-emitting GQDs were prepared from the acetylacetone after further microwave treatment which, for a longer time period, resulted in the appearance of green emission. Results suggested that increases in microwave treatment time and electronic coupling on the surface of -COOH groups took place and, thus, that this resulted in the formation of quasi-molecular fluorophores on the GQD surface [64]. Such a technique could be useful for composite preparation as well. Qiu et al. employed microwave irradiation to GQDs dissolved in ethylene glycol following the addition of cobalt nitrate hexahydrate, nickel nitrate hexahydrate and urea to the same. Microwave treatment for 10 min at 190 °C resulted in the formation of a GQD/Ni-Co LDH composite, which could be finally used as an electrode material for supercapacitors (Figure 7) [65]. Utilizations of carbohydrates [66], biomass [67], and amino acids [68] can also be found for the microwave assisted bottom-up synthesis of GQDs.

**Figure 8:**
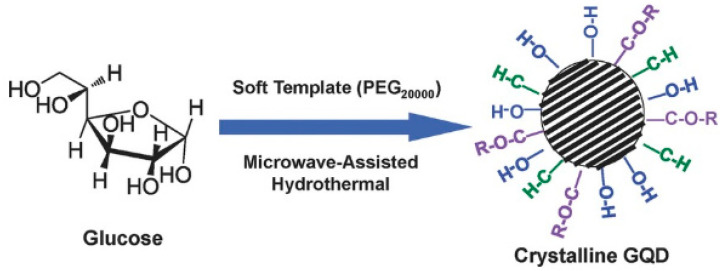
Preparation of the GQDs by a soft-template microwave-assisted hydrothermal method. Reprinted with permission from Ref. [77].

Additionally, the synthesis of GQDs via the soft-template method is common nowadays. This route helps to achieve very precise control over the size distribution of the particles, and complicated purification steps are mostly bypassed. However, aggregation of particles due to *π–π* stacking is common and cannot be avoided most of the time. Tang et al. synthesize monodispersed single-crystalline GQDs by using soft template method, followed by microwave and hydrothermal treatment to the starting materials (Figure 8) [77].

**Figure 9:**
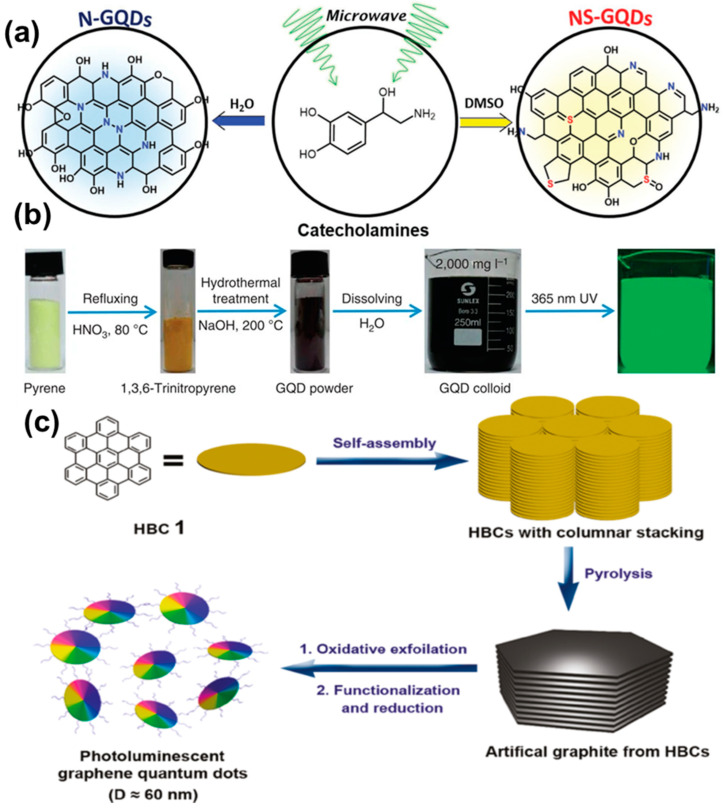
(**a**) Illustration for the microwave synthesis of N-GQDs and N,S-GQDs from a single precursor. Reprinted with permission from Ref. [61]. (**b**) Steps for the hydrothermal synthesis of colloidal GQDs. Reprinted with permission from Ref. [75]. (**c**) Schematic illustration for the synthesis of GQDs via the soft templet method. Reprinted with permission from Ref. [78].

The single precursor-derived rapid synthesis was demonstrated by Kim and co-workers, resulting in the doping of heteroatoms inside the GQDs structure (Figure 9a). It was found that the doping of heteroatoms could assist in improving the photocatalytic activity during the aerobic oxidative coupling of amines [61]. Li et al. optimized the microwave treatment of 1,3,6-trinitropyrene under alkaline conditions to produce high QY (35%) and single-crystalline GQDs with a few-layer structure. The synthesis process demonstrated here was rapid, and sufficient absorption could be observed in the visible region, which is desirable for optical applications [62]. Hydrothermal treatment of the precursor molecules has been found to be another effective way to produce GQDs. In a recent report, it was revealed that microwave post-treatment could result in reduced non-radiative relaxation, as confirmed by X-ray photoelectron spectroscopy (XPS) analysis. Thus, a significant enhancement in the emission intensity could be observed under a microwave environment [63]. In this method, citric acid has been used extensively by various groups as the carbon source for the preparation of GQDs [69,70,71,72]. During the synthesis, citric acid is reacted with another precursor molecule, leading to the generation of the graphitic core. In this regard, the utilization of urea, thiourea, and amine molecules is common [69,70,73,74]. Characterization reports suggest that hydrothermal treatment of those starting materials not only produced graphitic structures inside the core, but also resulted in hetero atom doping (N or S doping) inside the structure. Thus, fascinating optoelectronic properties have been generated. Polyaromatic hydrocarbons (PAHs) and amino acids have also been used to produce high QY GQDs. (Figure 9b) [75,76]. Wang et al. applied hydrothermal fusion to PAH molecules to produce single-crystalline GQDs under alkaline conditions. Here, the temperature varied between 90 and 200 °C. Such a technique offered high PL QY (23%), long-term photostability, and a sufficient molar extinction coefficient [75]. Guo et al. graphitized 1,5-dinitronaphthalene in aqueous ammonium solution via a one-pot hydrothermal method. They treated the starting materials in a Teflon-lined autoclave at 200 °C for 18 h. Particles with a 1.5 nm average diameter were formed at the end of the reaction and its optical properties were highly dependent upon the alkaline conditions [76]. The main drawbacks of this process are the time-consuming and costly synthesis process, along with tedious purification steps. However, ultimately, by applying such a technique, ultra-small particles are produced with novel fundamental properties, suitable for versatile applications.

Here, glucose was used as the carbon source, and polyethylene glycol (PEG_20,000_) was used as the soft template. During the treatment in presence of external radiation, a limited number of precursor molecules (glucose here) entered inside the PEG template and, subsequently, carbonization, nucleation, and growth of the particles took place. In this process, the size of the particles could be easily controlled simply by adjusting the duration of heating. In another study, hexa-peri-hexabenzocoronene (HBC) was utilized to prepare disk-like mono dispersed GQDs (Figure 9c) [78]. The novelty of this work was that the HBC molecules not only served as the carbon source during reaction, but they also acted as the template for GQDs synthesis. After the synthesis, the formation of homogeneous disk-like particles was confirmed by atomic force microscopy (AFM) images. Apart from polymers, the utilization of small molecules (such as citric acid and carbon disulphide) as the template has also been found to be useful for the bottom-up GQD synthesis [79,80].

A solution-chemistry method involves the integration of small molecules through multiple organic reactions. Yan et al. reported such a solution-chemistry-based process to generate stable GQDs with uniform shape and size [81]. By following such a technique, they were able to tune the number of conjugated carbon atoms and, thus, the size of the QD system. Oxidative condensation of polyphenylene dendritic precursors, prepared via stepwise solution-chemistry, led to the formation of fused graphitic moieties. By varying the number of conjugated carbon atoms and chemical functionalization to the surface, band gap and redox potentials can be tuned easily for device fabrication purposes. In another study, the same research group reported that the HOMO–LUMO energy gaps of colloidal GQDs, prepared via a solution-chemistry method, can be controlled by varying the size of the QD system (whereas their redox potentials can be tuned by applying surface functionalization to the particles) [82]. Thus, the solution-chemistry method has been found to be an important technique for GQD preparation in terms of device fabrication purposes.

## 3. Structure and Properties of GQDs

### 3.1. GQD Structures

**Figure 10:**
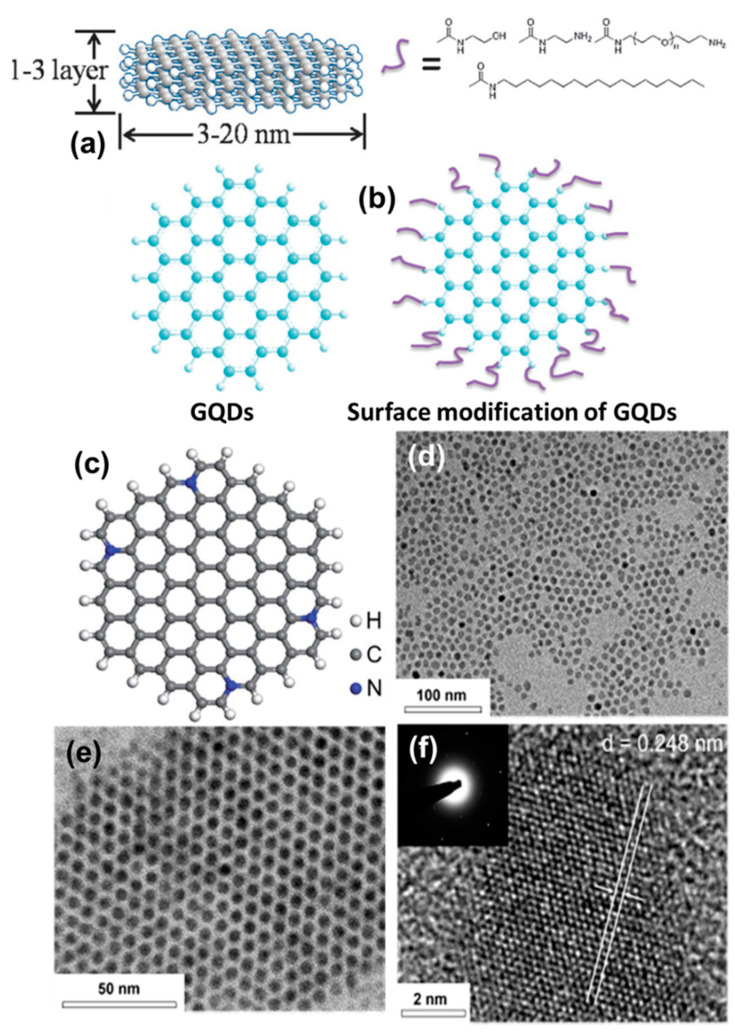
(**a**) Illustration for a few layers of stacked graphene in the QD system and (**b**) surface modification of colloidal GQDs with the help of ligands or functional groups. Reprinted with permission from Ref. [83]. (**c**) Optimized structure of a heteroatom doped GQDs. Reprinted with permission from Ref. [84]. Representative (**d**,**e**) TEM and (**f**) HRTEM images of GQDs. Reprinted with permission from Ref. [88].

As discussed in the previous section, GQDs can be synthesized from various precursors by using different synthesis approaches. Now, depending upon the process of synthesis, the inherent complex structures, and the nature of surface functional groups, the size and the shape of particles change [83]. Typically, GQDs consist of conjugated (sp^2^ hybridized) carbon atoms along with sp^3^ carbons, oxygen-related functional groups, and surface defects. Inside the core, a few layers of graphene remain present on top of each other, or in the lateral dimension (Figure 10a). On the other hand, various functional groups (such as carboxyl, carbonyl, epoxy, hydroxyl, amide, and amine) and ligands remain present over the surface of particles (Figure 10b) [83]. Recently, various research groups have demonstrated efficient strategies for doping heteroatoms inside the graphitic network (Figure 10c) [84,85,86,87]. The presence of heteroatoms such as N, S, P, or B has been found to produce fascinating optoelectronic properties, which we shall discuss in the following section. Their high crystallinity arises due to the honeycomb lattices (Figure 10d–f) of graphene, which can also be altered by means of heteroatom doping, thus creating structural defects, sometimes via surface functionalization. During the high-resolution transmission electron microscopy (HRTEM) study, d-spacing of 0.248 nm (100), 0.28 nm (020), and 0.35 nm (002) is common for GQDs, which can also be identified by powder X-ray diffraction (PXRD) analysis [88,89]. The extent of structural order and edge quality can be studied by calculating the intensity ratios between the D-band and G-band (*I_D_*/*I_G_*) for the in-phase vibration from the graphitic lattice. During the Raman spectroscopic measurement, the *I_D_*/*I_G_* value has been found to vary between 0.5 and 1 depending upon the extent of graphitization inside the core [90]. Moreover, an intense G-band corresponds to the presence of highly crystalline graphite structures inside the QD system.

### 3.2. Properties of GQDs and Their Roles in Supercapacitor Devices

#### 3.2.1. Electronic and Electrochemical Properties of GQDs

**Figure 11:**
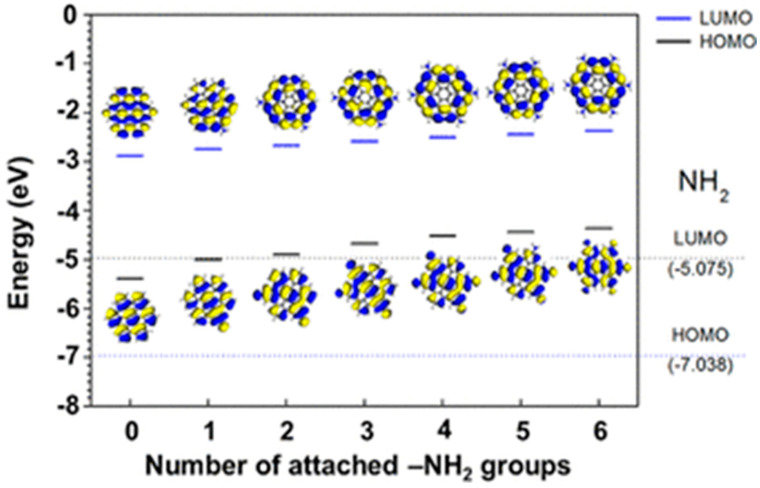
Systematic variations in HOMO–LUMO energy levels and the bandgaps due to increasing the number of attached -NH_2_ groups. Reprinted with permission from Ref. [105].

As discussed in the previous section, two-dimensional graphene consists of a repeated unit of honeycomb structures. They have several remarkable properties, among which charge mobility and high conductivity are worthy of mention. As found, graphene has two ‘conical’ points per Brillouin zone. A linear energy dispersion surges in the Dirac cone as the valence band and conduction band meet each other at the Dirac point. Such a linear energy dispersion results in massless carrier behavior, anomalous quantum Hall effect, and significant delocalization [91,92,93,94]. In 2008, Morozov et al. showed that the electron–phonon scattering in the bilayer graphene structure could be so weak that the carrier mobility in graphene exceeded over 200,000 cm^2^ V^−1^ s^−1^ under room temperature conditions [95]. Stormer and co-workers achieved an excess of 200,000 cm^2^ V ^−1^ s^−1^ mobilities at ∼2 × 10^11^ cm^−2^ electron densities by suspending monolayer graphene over the Si/SiO_2_ gate electrode [96]. High carrier mobility property has been reported by other groups as well [97,98,99,100]. Thus, due to its extraordinary electronic properties, nowadays graphene is considered the most promising candidate for future electronic devices. However, graphene is a zero-band gap semiconductor. Since there is no energy gap between the valance band and conduction band, the electrical conductance is nonzero even if there are no free charge carriers in the graphene layers [101]. Besides, quantum–mechanical confinement of the charge carriers is challenging, as the Klein tunnelling matches with the wave functions of positron and electron across the barrier [102,103]. During some of the device fabrication processes, the presence of a bandgap in the semiconducting layer is important. Therefore, to tune the band gap energy and strengthen the influence of charge transport phenomena, two-dimensional graphene has been transformed into zero-dimensional GQDs. Quantum confinement, surface effects, and conjugated carbon–carbon networks help to modulate their electronic and electrochemical properties. Peng et al. prepared GQDs from carbon fibers by following the acid oxidation technique. Here, they tuned the energy gap between the two levels by varying the reaction temperature [104]. Yan et al. tuned the band gap energy of GQDs by simply varying the shape and size of the particles [81]. The interlayer spacing in GQDs was found to be higher than that of the pristine graphite due to the presence of surface functional groups and doped hetero atoms. In another study, Jeon’s group reported that surface functional groups, and their electron donating or withdrawing nature, strongly affected the bandgap and, thus, the electronic properties of GQDs [105]. They showed that the functionalization of GQDs with electron-donating groups gradually reduced the energy gap due to charge redistribution (Figure 11). With increasing the number of electron-donating groups, the electron density in graphitic structures considerably increased, and this resulted in the bandgap reduction. Conversely, the electron-withdrawing nature showed the opposite trend. It is important to mention that tunning of the band gap energy or the surface groups strongly affects the charge transportation behavior of GQDs.

**Figure 12:**
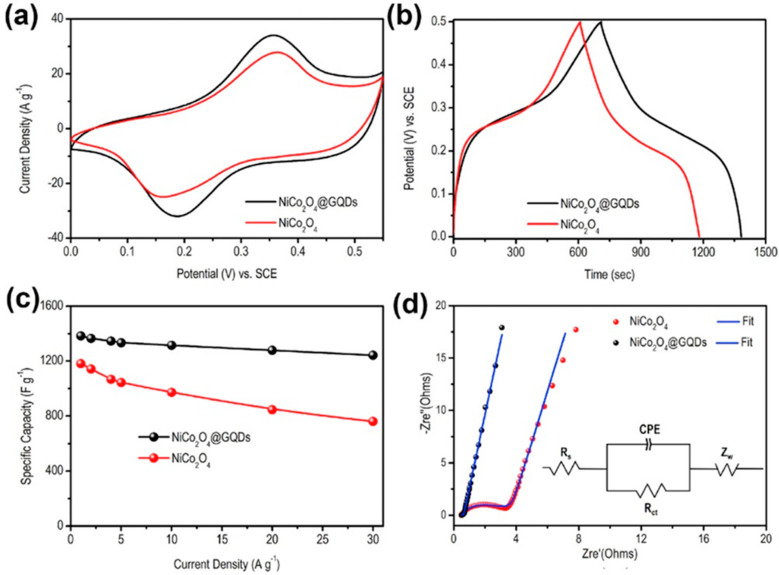
Electrochemical characterizations of the electrodes. (**a**) Cyclic voltammetry (CV) plot. (**b**) Galvanostatic charge-discharge (GCD) plots at 1 A g^−1^ current density. (**c**) Variations in specific capacitances at different current densities. (**d**) Fitted Nyquist plots along with the equivalent circuit diagram in the inset. Reprinted with permission from Ref. [107].

Tunable bandgap and high charge transport phenomena of GQDs have been successfully utilized to modify the electrode surfaces. The graphene structure inside GQDs boosts the electrical conductivity, electrolyte diffusion, and charge transportation between the electrode and analyte and, thus, leads to improved electrochemical performance. To date, various approaches have been reported to design GQD-based electrochemical devices with modified electrode surfaces and properties. N,S-GQDs were prepared via the hydrothermal method and, after that polymerization of aniline, resulted in the formation of N,S-GQDs/polyaniline (PANI) composite material. The addition of N,S-GQDs to the PANI-based electrode resulted in higher electrical conductivity to the double layer capacitor through extended electron delocalization due to the π–π interactions [106]. Here, doped hetero atoms in N,S–GQDs acted as trapping sites for the electrolyte ions. Besides, GQDs assisted in enhancing the surface area of the electrode material which led to more access to the electrolyte ions. Compared to the PANI electrode, the N,S-GQDs/PANI electrode showed a more rectangular CV plot and longer discharge time, indicative of better electrochemical phenomena and higher specific capacitance. At the 0.5 A g^−1^ current density, the maximum specific capacitance for the N,S-GQDs/PANI electrode was 645 F g^−1^, whereas the same for the pristine PANI electrode was measured to be 177 F g^−1^. In another work, Luo et al. conjugated GQDs with the NiCo_2_O_4_ p-type semiconductor, and the electrochemical properties were measured by using a three-electrode system in 2 M KOH electrolyte [107]. Here, a platinum electrode and a Ag/AgCl electrode acted as the counter and reference electrodes, respectively. As shown in Figure 12a, the GQDs/NiCo_2_O_4_ electrode consisted of a pair of enhanced redox peaks at 0.18 V- and 0.35 V-applied voltages. Such a peak originated due to good conductivity and fast charge transportation ability through GQDs/NiCo_2_O_4_ electrode material. Systematic variations in discharge time and specific capacitance have been shown in Figure 12b,c. The charge-transfer resistance (R_ct_) of the electrodes was studied by using electrochemical impedance spectroscopy (EIS). In Figure 12d, the Nyquist plots confirm 0.035 and 2.15 Ω of R_ct_ for GQDs/NiCo_2_O_4_ and NiCo_2_O_4_ electrodes, respectively. This result further shows the better electrochemical activity of GQDs incorporated electrode material in comparison to the pristine counterpart.

In another device configuration, N-GQDs were incorporated into cubic porous carbon via electro-deposition method by using a two-electrode system [108]. Deposition of N-GQDs in an aqueous solution was done over a porous carbon/carbon paper (CP) working electrode in presence of a Pt foil counter electrode. The process was continued for 3 h at 2 V for successful deposition of N-GQDs into the porous carbon. In this work, the hydrophilicity of the electrode material was checked by measuring contact angles. The contact angle for the composite electrode material was found to be 81.4°, and the same for the porous carbon-based electrode was measured to be 125.2°. This indicated that surface wettability was greatly improved after the incorporation of N-GQDs. The average pore size increased from 1.7 nm to 4.0 nm after the incorporation of N-GQDs into the porous carbon, which is beneficial for charge storage and quicker charge transportation. Higher discharge time, lower charge transfer resistance, and sufficient energy density for the N-GQDs-based electrode material were also confirmed from GCD curves, Nyquist plots, and Ragone plot, respectively. Thus, good conductivity and their ability to improve the electrochemical behaviors of electrode materials have added an extra dimension to the GQD research for developing high-performance supercapacitor electrodes.

#### 3.2.2. Specific Surface Area

Extensive surface area is one of the many intriguing properties of GQDs. An electrode material graphene faces significantly reduced surface area due to agglomeration or restacking of the sheets through π-π stacking or van der Waals interactions. On the other hand, although reduced GO (rGO) has been utilized for energy storage devices, relatively lower electrical conductivity limits their commercial applications. GQDs have emerged as a new kind of material by combining the exceptional properties of graphene and QDs and, thus, show an edge in terms of the fundamental properties as compared to the other graphene-based materials [109]. Gomes’s group applied chemical treatment, followed by ultrasonication, to few-layer graphene sheets after the treatment edge enriched activated GQDs were produced. They showed that, due to the presence of such activated edges, the specific surface area of GQDs was enhanced by many factors. As confirmed by the Brunauer–Emmett–Teller (BET) analysis, the specific surface area of layered graphene sheets and GQDs was 1289 m^2^ g^−1^ and 1502 m^2^ g^−1^, respectively. The higher surface area of GQDs not only assisted in the sufficient adsorption of ionic charges over the surface, but also the electrochemical performance could be improved remarkably [110]. Zhang et al. prepared GQDs from GO powder via hydrothermal route. BET analysis in this study confirmed a larger specific surface area of the as-produced GQDs as compared to the other graphene-based materials [111]. Additionally, several reports indicate improved specific surface area of the electrode material following the incorporation of GQDs. For example, Zhao et al. prepared an activated carbon nanofiber fabric-based composite with uniformly embedded GQDs (termed AGRCNF) [112]. The AGRCNF composite showed good conductivity, improved mechanical properties, and a very high surface area following the reinforcement of GQDs. As confirmed, the measured surface area for carbon nanofiber fabric (CNF) was only 140 m^2^ g^−1^. However, for AGRCNF, the same was measured to be 2032 m^2^ g^−1^. Such a high surface area of the composite material finally helped in achieving good specific capacitances. In another work, Zhang et al. showed that GQDs can significantly improve the specific surface area and packing density of ultra-microporous carbons, which facilitated fast charge transport kinetics [113]. Thus, enhanced gravimetric and volumetric capacitances were achieved. A similar trend has been reported by other research groups as well [114,115,116,117]. In reality, a larger surface area offers exposed edges, more active sites, and better contact between the electrode material and the electrolyte (suitable for charge–carrier adsorption and diffusion).

#### 3.2.3. Roles of Doped Hetero Atoms and Surface/Edge Groups

**Figure 13:**
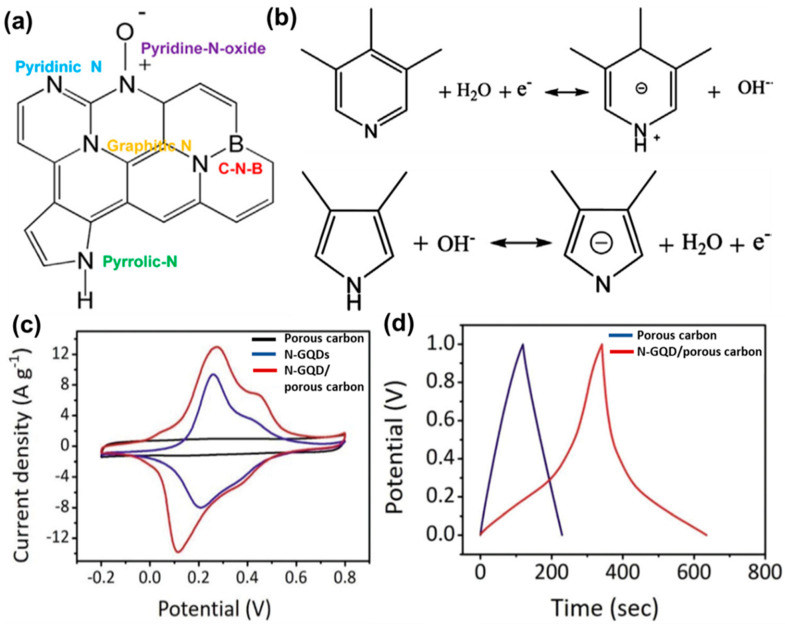
(**a**) Schematic representation for the hetero atom doped graphitic nano structure. (**b**) Proposed pseudocapacitive reactions for the nitrogen-containing species. Reprinted with permission from Ref. [121]. (**c**) Cyclic voltammetry plots for porous carbon, N-GQDs, and N-GQDs/porous carbon electrodes at 10 mV s^−1^ scan rate and in 1.0 M H_2_SO_4_ electrolyte. (**d**) Galvanostatic charge–discharge plots for the supercapacitor electrodes at 0.5 A g^−1^ current density. Reprinted with permission from Ref. [108].

Various functional groups, present over the GQD surface, have been found to induce a positive effect on the charge storage phenomenon. As reported, oxygen and nitrogen-containing groups provide large pseudocapacitance and improved wettability. Such pseudocapacitance arises due to Faradaic redox reactions by those surface functional groups in the ionic–liquid electrolyte. Yan’s group prepared a nanocomposite by coupling GQDs with MnO_2_ nanosheets [118]. The introduction of GQDs resulted in the generation of pseudocapacitance, contributing to the total capacitance of the electrode material. Thus, an improved charge storage phenomenon in the ionic–liquid electrolyte was observed. Here, the pseudocapacitive behavior of the electrode material was confirmed by cyclic voltammetry (CV) and a charge–discharge experiment. Li et al. developed N-GQDs—carbonfiber hybrid material for fabricating a flexible fiber-based supercapacitor electrode [119]. In this study, they showed that the presence of active oxygen-containing groups and pyrrolic-N/pyridone-N in the carbonaceous structure significantly enhanced the charge storage performance by inducing additional pseudocapacitance. Such an effective pseudocapacitance originated due to the Faradaic interactions between the surface functional groups and ions. The specific volumetric capacitance was measured to be 93.7 F cm^−3^ at a 20 mA cm^−3^ current density, seven times higher than that of a carbonfiber electrode [120]. Boron was doped by adding 1.0 g of boric acid powder into 0.1 g of GQDs, and the resultant mixture was allowed to sonicate under vigorous stirring. Carbonization of the resultant fine powder at 800 °C for 1 h under an N_2_ environment produced boron, nitrogen (B, N), and co-doped graphitic carbon nanosheets [121]. A pair of redox peaks were identified in the CV plot, and a pseudocapacitive contribution as high as 48% was measured at 10 mV s^−1^ for the electrode material. However, for the undoped material, such contribution was found to be less. Li et al. confirmed that B and N act as active sites for such pseudocapacitance. Possible Faradaic redox reactions for the nitrogen species have been shown in Figure 13a,b. As mentioned, such reactions involve proton exchange of the electrochemically active functional groups through the proton-coupled electron transfer (PCET) mechanism. In the case of boron, although it is known that it shows electrochemical activity for the pseudocapacitive phenomenon, the appropriate mechanism needs to be explored further. Such redox reactions not only generate the pseudocapacitive behavior, but also boosts the ion adsorption, electronic charge density, and double-layer capacitance of electrodes. In another study, N-GQDs were incorporated inside the porous carbon material to improve the energy densities of supercapacitor electrodes [108]. In this study, CV analysis (Figure 13c) suggested an enhanced capacitive response of the N-GQDs/porous carbon-based electrode due to the higher electric double layer capacitance (EDLC) and an additional contribution from the pseudocapacitance. Furthermore, the recorded galvanostatic charge–discharge plot was distorted from the triangular shape and consisted of a small plateau (Figure 13d). Such deviation from the linearity confirmed the pseudocapacitive behavior as well. For the N-GQDs/porous carbon material, the maximum specific capacitance was measured to be 780 F g^−1^ at a 10 mV s^−1^ scan rate, and the same for the porous carbon material was measured to be only 188 F g^−1^. Researchers surmised that N-GQDs possessed substantial pseudocapacitive activity, and improved electrolyte wettability, due to the doped nitrogen functional groups. An enhanced wettability assisted in better diffusion of the electrolyte throughout the electrode material. Thus, overall charge storage performance was improved. Similar effects have been reported in the case of GQDs/carbon black [122], GQDs/carbon fiber [123], GQDs/TiO_2_ nanotube [124], GQDs/polyaniline nanofiber [125], and GQDs/CNT/carbon cloth [126] composites. Table 1 represents some recent reports on the changes in specific surface area and series resistance (R_s_), or the charge transfer resistance (R_ct_), due to incorporation of GQDs inside the composite electrode materials.

## 4. Energy Storage Applications

**Figure 14:**
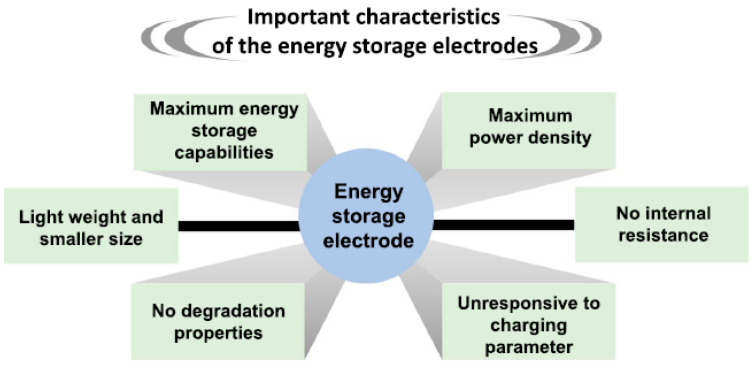
A few important characteristics of the energy storage electrodes. Reprinted with permission from Ref. [8].

The prior research clearly demonstrated that the material’s characteristics, such as the maximum energy storage capability, high power density, low internal resistance, insensitivity to charging parameters, no degradation, small size, and light weight, could be key parameters for developing new energy storage devices (Figure 14).

### 4.1. Batteries

**Figure 15:**
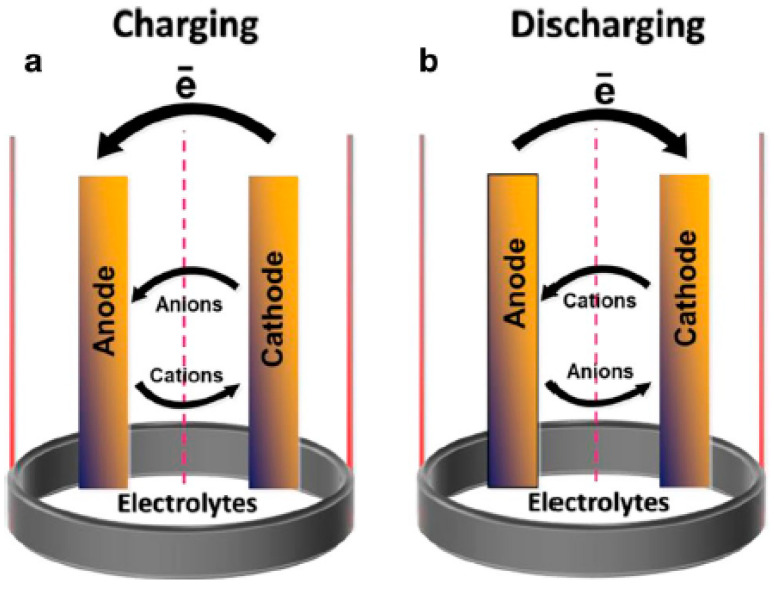
Schematic illustration with general mechanism of (**a**) charging and (**b**) discharging process in batteries. Reprinted with permission from Ref. [100].

Batteries are energy storage devices that store energy in the form of chemical energy and, consequently, convert it into electrical energy via redox reactions (Table 2) [127,128,129,130,131,132,133,134]. They are broadly classified into two categories: the primary cell and the secondary cell. In the primary cells, the chemical reactions are unidirectional. A primary cell cannot be used for the second time once the chemicals are consumed completely. In a secondary cell, charges can be restored by passing a current through the two oppositely charged electrodes. Typically, such secondary cells are constructed with two electrodes, an electrolyte, and a separator. The oxidation occurred over the positive electrodes, whereas the negative electrodes accept the electrons through the reduction process, which is displayed in the Figure 15 and equation. These charges travel through the electrolytes over the external circuit and produce currents. These can be understood via the following reactions that occur during the charge/discharge process.

Charging process

Anodic reaction

2Li → 2Li^+^ + 2e^−^ (oxidation, electron loss)

Cathodic reaction

S + 2Li^+^ + 2e^−^ → Li_2_S (reduction, electron gain)

Discharging process

2Li + S → Li_2_S (overall electrochemical reaction)

**Figure 16:**
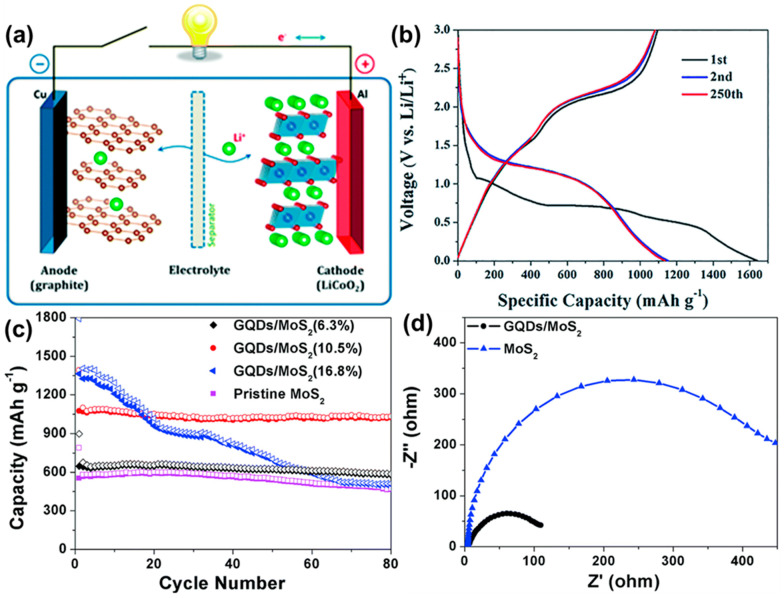
(**a**) Schematic representation of a LIB working principle. Reprinted with permission from Ref. [139]. (**b**) Charging-discharging profiles in different cycles of the NiO@Co3O4@GQDs electrode in LIB. Reprinted with permission from Ref. [130]. (**c**) Effect of GQDs concentration on the cyclic performance of LIB electrode. (**d**) EIS spectra. Reprinted with permission from Ref. [131].

So far, different types of secondary cells are constructed, such as lead–acid, nickel–metal–hydride, cadmium batteries, sodium ion, lithium-ion batteries and so on [135,136,137,138,139]. Among them, lithium-ion batteries (LIBs) (Figure 16a) are being widely used in portable electronic devices and electric vehicles nowadays. They provide high specific capacitance, good cycling stability, and sufficient energy density. In recent years, in order to improve their performances, GQDs have been successfully employed as active materials in such LIBs. Wang’s group developed multilayer NiO@Co_3_O_4_ composite material modified with the GQDs and used it as an anode material for LIB [130,140]. Incorporation of GQDs resulted in excellent electrochemical behavior. Figure 16b represents the charging–discharging profile of the electrode at the 1st, 2nd, and 250th cycle at 0.1 A g^−1^ current density. Two plateaus (∼1.1 V and ∼0.7 V) could be observed, which were attributed to the lithiation process in Co_3_O_4_ and NiO, respectively. After the incorporation of GQDs, reduced interfacial resistance and higher surface area were found due to the –COOH functional groups present over GQDs particles. Additionally, the surface functional groups in GQDs showed high affinity to the Li^+^ ions. As an anode material for LIB, the composite showed 1158 mA h g^−1^ of reversible capacity at 0.1 A g^−1^ and maintained good cyclic stability. In another study, Wang et al. encapsulated GQDs inside the hollow porous SiO_2_ via the self-assembly method [141]. They performed CV analysis at various scan rates, capacity distributions, and surface-controlled pseudocapacitive behavior analysis to prove that Li^+^ storage and Li^+^ transfer were facilitated by abundant heterointerfaces, generating a local electrical field from GQDs to the hollow SiO_2_. A maximum ∼2250 mA h/g specific capacitance at 0.2 A g^−1^ current density was measured for the GQDs/SiO_2_. It was revealed that doped heteroatoms in GQDs could also assist in improving the specific gravimetric capacity of LIBs. For example, B-GQDs and N-GQDs were separately mixed with acetylene black and sodium carboxymethylcellulose (CMC) in water, and the resultant slurry was coated over the copper foil to prepare the working electrode. 1 M LiPF_6_ was used as the electrolyte. The charge transfer resistance value for each electrode material was calculated by using the EIS study. The R_ct_ value for the only GQD-based electrode was found to be 201 Ω. However, the same for N-GQDs and B–GQD-based electrodes were found to be 106 and 194 Ω, respectively, under similar experimental conditions. Researchers inferred that physical adsorption followed by diffusion of ions was facilitated by heteroatoms, resulting in lower R_ct_ and improved specific gravimetric capacitance [142]. Transition metal fluorides are another fascinating class of electrode materials with the merits of low costs, high voltage, and high specific capacity. Currently, attempts have been made to improve their performances in the LIBs. In recent work, vertical nanosheets of iron fluoride surfaces were modified via electrophoresis of GQDs. It was found that integration of GQDs not only resulted in enhanced electrical conductivity but, also, that the electrochemical and cycle performance were greatly enhanced [143]. Guo et al. introduced GQDs inside the two-dimensional molybdenum disulfide (MoS_2_) sheets to prepare a GQDs/MoS_2_ composite material [131,144]. In this study, they used the GQD/MoS_2_, added to the conductive material and polyvinylidene fluoride (PVDF), as a working electrode. A lithium sheet and a Clegard 2300 microporous film were used as a negative electrode and a separator, respectively. LiPF_6_ in a mixture of ethylene carbonate, dimethyl carbonate, and ethyl methyl carbonate (1:1:1 in volume) was used as the electrolyte. It was proved that the introduction of GQDs inside the two-dimensional structure not only enhanced the conductivity but, also, boosted the lithium storage ability. Figure 16c represents the effect of GQD concentrations over the electrode cyclic performances. With an increase in the GQD concentrations from 6.3 to 10.5%, a significant rise in the discharge capacity and capacity retention could be observed. An initial discharge capacity of 1393 mA h g^−1^ was achieved at 10.5% of GQDs concentration, and 94% of this was retained after 80 cycles of use. At 16.8% of GQD concentration, although 1794 mA h g^−1^ discharge capacity was recorded, the cyclic stability of the electrode material reduced significantly. These results suggested that the highest efficiency of the electrode material could be achieved at a moderate concentration of GQD in the electrode material. Figure 16d shows the EIS plots for a pristine MoS_2_ electrode and GQDs-modified MoS_2_ electrode. From this plot, the R_ct_ for the GQD/MoS_2_ electrode was measured to be 109 Ω, which was much lower than that of the pristine MoS_2_ electrode (R_ct_ = 447 Ω). The result suggested improved electrochemical lithium storage kinetics and better rate performance due to the incorporation of GQDs.

**Figure 17:**
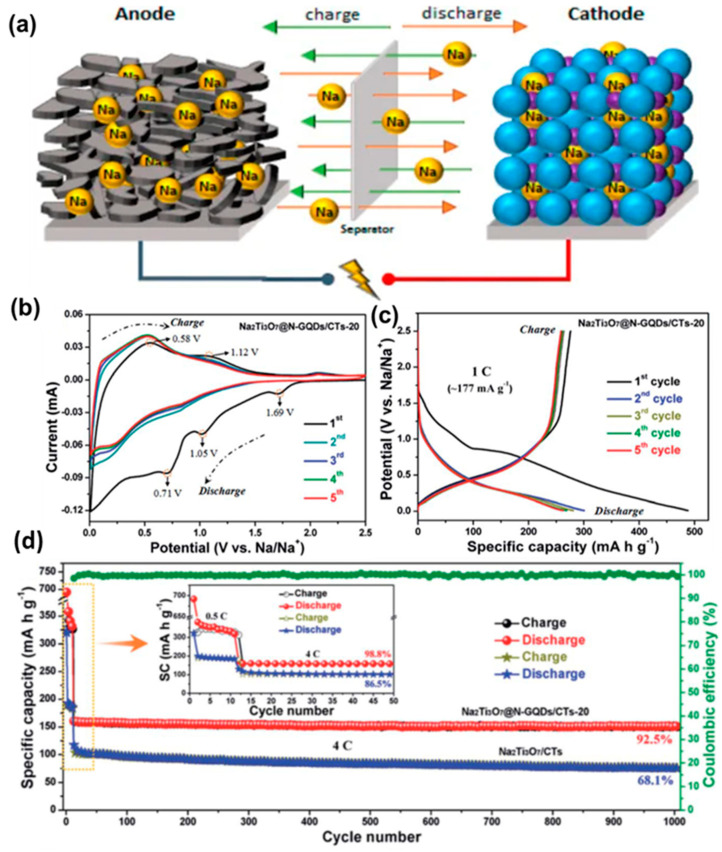
(**a**) Schematic representation of a SIB working principle. Reprinted with permission from Ref. [146]. (**b**) CV curves for the Na_2_Ti_3_O_7_@N-GQDs/CT electrode in different cycles. (**c**) GCD plots at a current density of 1C. (**d**) A comparison between the cycling performances of the Na_2_Ti_3_O_7_@N-GQDs/CT and Na_2_Ti_3_O_7_/CT electrodes. Reprinted with permission from Ref. [148].

Due to their widespread use, some surveys suggest that Li demand is expected to be as high as 900 ktons per year in 2025 [145]. Li is not an earth-abundant element. Consequently, a significant jump in its price is expected depending upon its high demand and limited supply. Therefore, as an alternative to LIBs, sodium-ion batteries (SIBs) have attracted the research community. Sodium is a naturally abundant element, and the corresponding devices are low-cost, efficient and stable. The working principle of a SIB is depicted in Figure 17a, which is quite similar to the LIB [146]. So far, various materials have been studied to construct efficient SIBs, and the utilization of GQDs is notable. VO_2_ is one of the high-capacity and mostly used electrode materials for SIBs. However, they are relatively less stable. Chao et al. prepared a novel binder-free cathode material by growing VO_2_ over the graphene networks [147]. Subsequently, the coating of GQDs onto the VO_2_ resulted in additional protection to the VO_2_ surface and boosted the electrochemical properties as well. A good Na storage capacity of 306 mAh/g was achieved at 100 mA g^−1^, along with 110 mAh/g of capacity at 18 A g^−1^, after 1500 cycles of use. As cathode material, the outstanding electrochemical properties of VO_2_ are related to long-range, single-crystallinity exposed facets and large interlayer spacing. Furthermore, functionalization with GQDs made the surface more lipophilic which facilitated better penetration of Na^+^ ions and, thus, boosted the reaction kinetics. Layered Ti-based compounds have been used extensively as promising materials for SIBs because of suitable operating voltage and very little structural expansion. Low-cost and stable anode materials with high energy density have been reported by using these layered structures. Results suggested that further modification with GQDs improved the Na^+^/e^−^ transportation and charge/discharge rates. In a recent study, N-GQDs have been decorated over an array of vertically aligned Na_2_Ti_3_O_7_ nanofibres in order to produce ultra-stable and high-rate SIBs [148]. Figure 17b represents the CV curve for N-GQD-integrated Na_2_Ti_3_O_7_ nanofibres/carbon textile (CT) electrodes (termed as Na_2_Ti_3_O_7_@N-GQDs/CT) in the different cycles. Here, in the first cycle, the broad peak appearing between 1.1 V and 0.01 V was attributed to Na^+^ diffusion and generation of a solid electrolyte interface (SEI) layer. However, in the second cycle, the broad peak (~1.1 V) disappeared, indicating structural changes due to the electrochemical reactions. Improved reversibility could be observed from the third cycle, implying a stable sodiation/desodiation process. Figure 17c shows the GCD plots in different cycles at a rate of 1C. The initial discharge capacity was measured to be ~488.0 mA h g^−1^, much higher than the same for the Na_2_Ti_3_O_7_/CT electrode. The cycling performance of the materials has also been presented in Figure 17d. As found, after 1000 cycles of use, the Na_2_Ti_3_O_7_@N-GQDs/CT could retain ~92.5% of its initial efficiency. However, for the Na_2_Ti_3_O_7_/CT electrode, the measured value was 68.1% after 1000 cycles (Figure 17d). In another study, Deng et al. shielded Na_3_(VO)_2_(PO_4_)_2_F@C nanocuboids with GQDs to improve the capacity and rate performance of electrodes [149]. Recently, a theoretical study suggested that the nature of defects and net surface charge can have a significant effect on the extent of Na^+^ adsorption [150]. Although SIBs have shown some initial promises for the replacement of LIBs, more investigations are still required to achieve performances similar to LIBs. Herein, GQDs are expected to play a crucial role in improving battery performance in the future.

### 4.2. Supercapacitors

**Figure 18:**
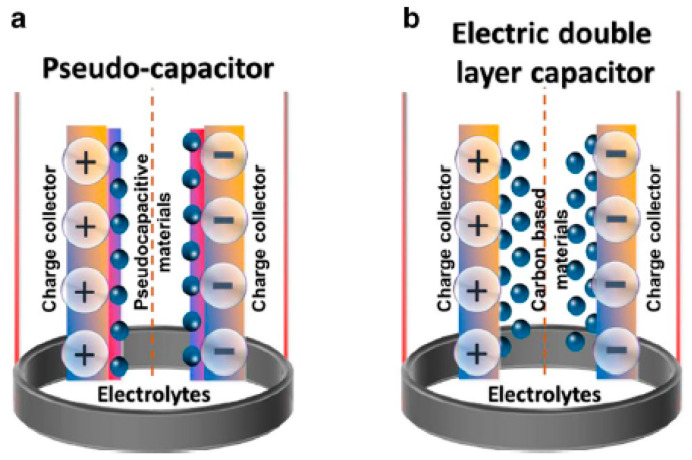
Schematic illustration with general mechanism of (**a**) a pseudocapacitor and (**b**) an electric double layer capacitor. Reprinted with permission from Ref. [8].

Supercapacitors are high-capacity electrochemical capacitors where energy is stored either by using the electrochemical double layer (EDL) effect or by rapid surface redox reactions. In recent years, they emerged to be a prominent class of energy storage devices due to their fast charging and discharging, sufficient cyclic stability, and high-power density [151,152]. Furthermore, the development of novel electrode materials at the nanoscale and advanced device construction procedures have significantly improved their energy densities (sometimes closer to the batteries as well) [153]. Due to these superiorities, supercapacitor devices are often integrated with batteries or fuel cells for high-power energy harvesting applications [154]. Typically, supercapacitors consist of two parallel electrodes separated by ion permeable non-conductive material infused with electrolytes. Based on the charge storage mechanism, they can be broadly separated into three categories (Figure 18): (1) electric double-layer capacitors (EDLC), (2) pseudocapacitors, and (3) hybrid capacitors [155,156].

**Figure 19:**
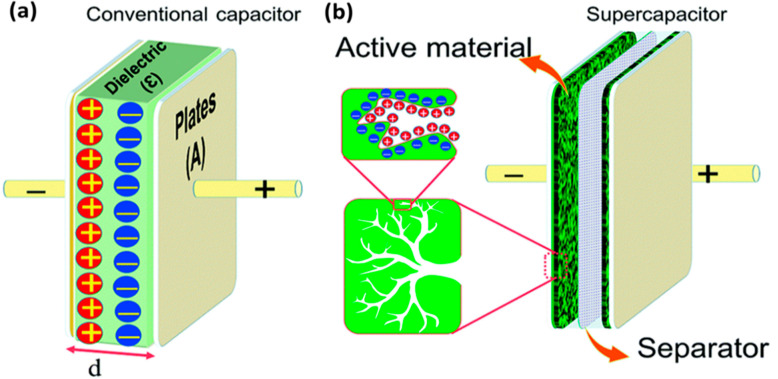
A schematic representation of (**a**) a conventional capacitor and (**b**) a supercapacitor along with the electrical double layer formation. Reprinted with permission from Ref. [157].

In an EDLC, the charge is stored in the Helmholtz double layer at the interface of electrode and electrolyte. Whereas, in a pseudocapacitor, the charge is electrochemically stored via redox reactions. In a hybrid capacitor, the charge can be stored electrostatically and electrochemically with the help of asymmetric electrodes [155,156]. Figure 19 represents the schematics for a conventional capacitor and supercapacitor. Conventional capacitors consist of two oppositely charged parallel plates separated by a thin insulating material, also called the dielectric layer (Figure 19a). Here, charge is stored as electric potential energy. In a supercapacitor, as shown in Figure 19b, charge is stored either by charge separation at the Helmholtz double layer at the electrode–electrolyte interface, or via Faradaic charge-transfer.

Carbon-based materials are attractive for the supercapacitor electrodes due to the high surface area, porosity, sufficient conductivity, abundant active sites, and, finally, because they are sustainable materials [157,158,159,160]. In this regard, GQDs have drawn a major attraction to the scientific community and detailed studies are being carried out on their utilizations in different types of supercapacitors. This section of the review article will demonstrate some recent advances in developing high-performance electrode materials and utilization of GQDs in improving device efficiencies.

**Figure 20:**
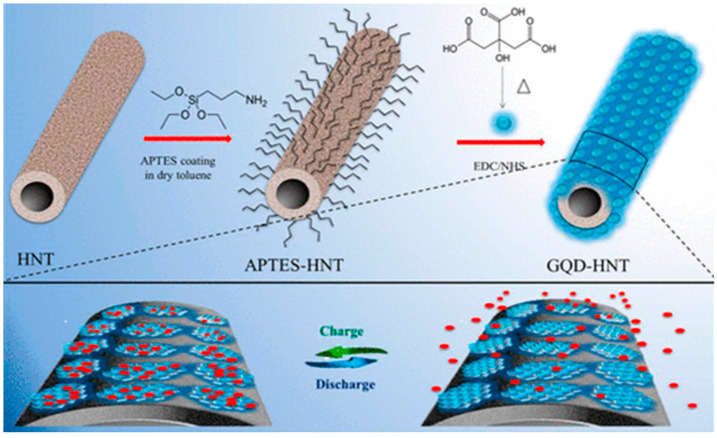
Synthesis of GQD-HNT composite electrode material for supercapacitor applications. Reprinted with permission from Ref. [162].

By using GQDs, Zhang et al. prepared hierarchical porous carbon nanosheets (HPCNs) to develop supercapacitor electrodes. The HPCNs consisted of loose-stacked internally connected graphene-like structures and a very high specific surface area of 1332 m^2^ g^−1^. The pore size distribution, good conductivity, abundant ion migration channels, and active sites made HPCNs suitable electrode material as well. A maximum specific capacitance of 230 F g^−1^ was achieved at 1 A g^−1^ current density with 74% of capacitance retention after 1000 cycles of use [161]. Halloysite nanotubes (HNTs) are naturally occurring clay materials and, very recently, their surface has been modified with GQDs to prepare electrodes for supercapacitors. Doong’s group coated the HNT surface with (3-aminopropyl)-triethoxysilane (APTES) and subsequently factionalized the surface with GQDs via EDC/NHS coupling reaction. The amide linkages, present inside the conjugated structure, provided more active sites for charge transportation (Figure 20) [162].

Tian et al. prepared highly conductive porous carbon (CPC) material via micelle-induced assembly of crystallized GQDs precursor and block copolymer soft templates [163]. Here, the interconnected mesoporous structure of GQDs, and high specific surface area of the material, assisted in fast ion transportation at high mass loading concentrations. In a three-electrode system, it showed 315 F g^−1^ specific capacitance at 1 A g^−1^ current density, and 6.45 Wh kg^−1^ energy density at 20 mg cm^−2^ mass loading. A transparent and flexible chelate of GQDs and monolayer graphene acted as an efficient electrode material for supercapacitors. Lee et al. electrophoretically deposited GQDs over graphene with the assistance of chelating metal ions. By using the composite material, a micro-supercapacitor was finally developed, which showed high energy storage performance under severe bending conditions [164].

**Figure 21:**
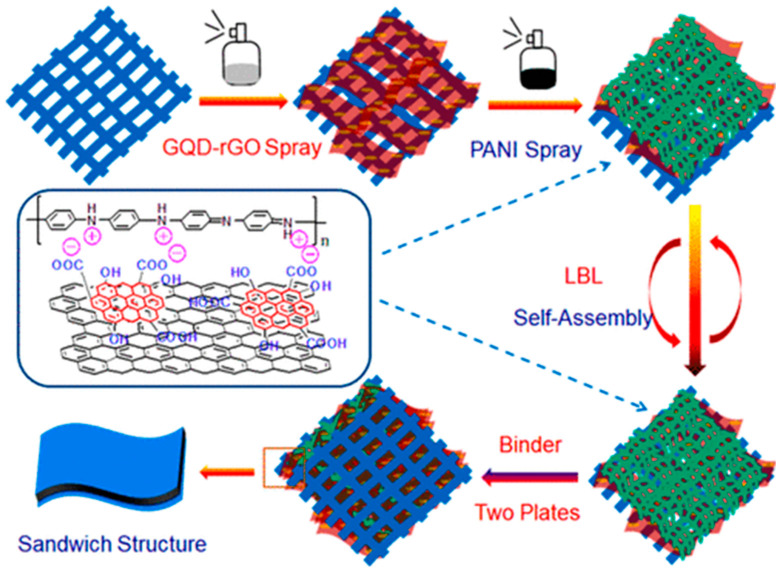
Layer-by-layer assembly of GQD-rGO and PANI over CFC for the formation of a supercapacitor electrode. The inset picture shows the interaction between GQD-rGO and PANI. Reprinted with permission from Ref. [169].

Recently, various conducting polymers have been used as electrode materials, as they offer good conductivity [165,166]. However, in terms of film stability and cyclic performance, they are not as good as carbon-based electrode materials. Thus, to improve the performance of such polymer-based electrodes, GQDs have been integrated. For example, a GQD-modified PANI composite (GQD-PANI) was prepared via chemical oxidation of aniline in the presence of ammonium persulphate (APS). After synthesis, the GQD-PANI composite was applied for energy storage applications. A maximum of 1044 F g^−1^ was achieved at 1 A g^−1^ current density with ~80.1% of capacitance retention after 3000 cycles of use. In the case of pristine PANI, maximum specific capacitance was found to be 206 F g^−1^ at 1 A g^−1^ current density [167]. In another work, N and S co-doped GQDs were integrated inside PANI to fabricate a symmetric capacitor [106]. The combination of pseudocapacitive and EDLC behavior of the electrode material was confirmed from rectangular-shaped CV curbs with broad humps. The device delivered an energy density of 17.25 Wh kg^−1^ at 500 W kg^−1^ power density. In devices, the enhanced energy storage performances were attributed to the doped hetero atoms in GQDs, which improved the electrical conductivity and captured more electrolytes [106,168]. Wang et al. prepared a carbon fiber cloth (CFC)-based supercapacitor by performing layer-by-layer self-assembly of GQDs-rGO and PANI over the CFC cloth (Figure 21) [169]. Such layer-by-layer deposition turned the CFC surface sufficiently hydrophilic in nature and improved the interaction between CFC and PANI without damaging the CFC’s internal structure. Here, the ionic groups over the GQD surface provided very good hydrophilicity to CFC and rGO induced sufficient electrical conductivity. By using H_2_SO_4_/polyvinyl alcohol (PVA) electrolyte gel, a maximum of 1036 F g^−1^ capacitance was achieved for PANI/GQDs-rGO/CFC-based symmetrical capacitor. Furthermore, in order to provide sufficient mechanical stability to the PANI films, researchers have blended PANI with other organic polymers. In a recent study, researchers have prepared a polymer nanocomposite of PANI-GQD blended with polyacrylonitrile (PAN) polymer [113]. In the composite material, GQDs provided good conductivity, enhanced electrolyte infusion, and improved current density. PAN, on the other hand, induced sufficient mechanical and chemical stability. There are several studies where GQDs have also been incorporated into other polymers such as polypyrrole (PPy), poly(3,4-ethylenedioxythiophene), and PVA for the superior charge storage phenomena [170,171,172,173].

**Figure 22:**
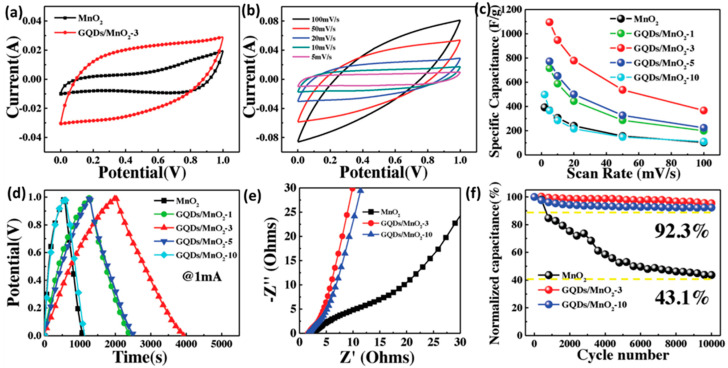
(**a**–**f**) Improvement in the electrochemical performance of MnO_2_-based supercapacitor electrode with the help of GQDs. Reprinted with permission from Ref. [174].

Heterostructural nanosheets were prepared by combining GQDs with transition metal oxides to develop high-performance supercapacitor electrodes. Jia et al. utilized GQDs to modify the intrinsic conductivity and specific surface area of MnO_2_. GQDs were attached to the MnO_2_ surface via Mn–O–C covalent bonds by a plasma-enhanced chemical vapor deposition (PECVD) process [174]. Integration of GQDs enlarged the operational voltage from 0–1 to 0–1.3 V and significantly improved the capacitive performance. Figure 22a–f represents the improvement in the electrochemical performance of MnO_2_ due to the integration with GQDs. In this work, a maximum specific capacitance of 1170 F g^−1^ was reported for the heterostructural electrodes along with the minimum R_ct_ values (2.5 for MnO_2_ and1.45 Ω for GQDs/MnO_2_).

Zhang et al. deposited MnO_2_-GQD over porous wood carbon (PWC) to produce advanced electrodes for supercapacitors. Here, PWC was used as a conductive matrix over which MnO_2_ and GQDs were deposited by the hydrothermal method. GQDs boosted the ion transfer throughout the electrode material, and simultaneously acted as a protective layer for MnO_2_. Compared to the PWC/MnO_2_ electrode, the PWC/MnO_2_-GQD electrode showed better electrochemical performance with 2712 mF cm^−2^ areal capacitance at 1.0 mA cm^−2^ current density and 95.3% of capacitance retention after 2000 cycles of use [175]. Similarly, GQDs have also been integrated into V_2_O_5_, RuO_2_, Fe_3_O_4_, and MoS_2_ to improve their electrochemical performance and accomplish improved charge storage phenomena [176,177,178,179].

**Figure 23:**
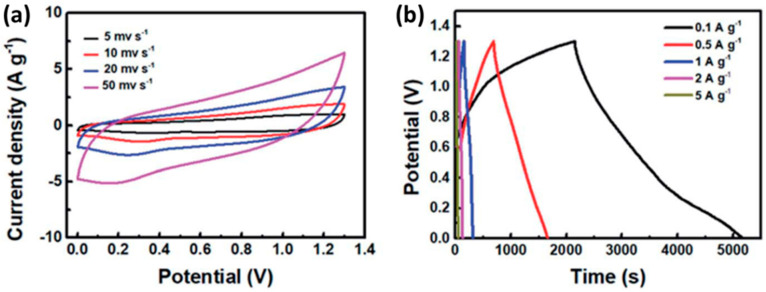
(**a**) CV plots of the asymmetric capacitor at different scan rates. (**b**) GCD plots for GQD/MnCo_2_O_4.5_/rGO supercapacitor at different current densities. Reprinted with permission from Ref. [182].

Binary metal oxides or sulfides are considered important classes of supercapacitive materials owing to sufficient capacity, fascinating morphology, high energy density, large specific surface area, and pore size distribution. In this section, we shall discuss some of the important efforts that have been made to improve their performance by using GQDs. Nickel aluminate (NiAl_2_O_4_) is a good alternative to develop biocompatible, robust, and low-cost electrode materials. NiAl_2_O_4_ contains Ni(II), which can be oxidized to Ni(III), giving rise to electrochemical potentials. To date, various additives, such as CNTs, graphene, and GO, have been blended into such binary metal oxides to improve electrical conductivity. Hryszko’s group prepared a GQD/NiAl_2_O_4_ composite by reacting crystalline nickel aluminate with citric acid at 200 °C under vigorous stirring [180]. After that, they utilized the GQD/NiAl_2_O_4_ composite to modify Au/carbon paste (CP) electrodes. The Au/CP/GQD/NiAl_2_O_4_ electrode showed higher current and improved specific capacitance, as compared to the Au/CP/NiAl_2_O_4_ electrode. In another work, GQDs were blended with CuMnO_2_ nanocrystals through the hydrothermal method to improve specific capacitance and efficiency [181]. Here, an asymmetric capacitor was developed by using activated carbon and a GQD/CuMnO_2_ nanoporous electrode as the negative and positive electrodes, and polyethylene paper as the separator. Zhang et al. induced GQDs inside MnCo_2_O_4,5_ to prepare a metal oxide/carbon composite for the supercapacitor application [182]. After the introduction of GQDs, nanoneedle composites were prepared. The GQD/MnCo_2_O_4,5_ composite material consisted of porous structures and highly conductive networks that provided excellent charge transfer phenomena and improved electrochemical performances. Finally, by using GQD/MnCo_2_O_4,5_ and rGO electrodes, an asymmetric capacitor was developed. The CV curves of the GQD/MnCo_2_O_4,5_/rGO supercapacitor under the different current densities are shown in Figure 23a. Rectangular shapes at relatively higher current densities indicated good capacitive behavior. A maximum capacitance of 200 F g^−1^ at 0.1 A g^−1^ current density was calculated from Figure 23b. However, improvement in the capacitive behavior could be found up to certain limiting concentrations of GQDs. Addition of an excess amount of GQDs resulted in structural changes in electrode material that restricted the ion movements.

Wang et al. improved the energy storage performance of NiCo_2_S_4_ by conjugating it with tryptophan-functionalized GQDs (Trp-GQDs) [183]. Trp-GQD were hybridized with NiCo_2_S_4_ by using a one-step hydrothermal process. Although NiCo_2_S_4_ showed very good electrical conductivity and high-power density, it suffered from structural instability, leading to poor cycling stability. In this work, they assembled a Trp-GQD/NiCo_2_S_4_ electrode as the positive electrode, activated carbon as the negative electrode, and cellulose filter paper soaked with 3.0 M KOH as the separator. The Trp-GQD modified NiCo_2_S_4_ electrode offered higher specific capacitance at all the current densities as compared to the NiCo_2_S_4_ electrode. Here, the improved capacitance was attributed to electrochemical synergy between Trp-GQD and NiCo_2_S_4_. Due to the adjacent redox peak potentials, the electrode reactions were promoted by each other during the electrochemical reactions. Additionally, high conductivity of Trp-GQD accelerated the electron transportation through electrode material and the binder layer. Their high polarity due to surface groups generated a strong affinity to the electrolyte ions, resulting in rapid ion transportation. Finally, a morphology study suggested that incorporation of GQDs improved the mechanical property of the binder layer. Sufficient elasticity and good mechanical strength ensured better structural stability of the electrode material, leading to outstanding cyclic performance [183]. It was reported that decoration of molybdenum (Mo) particles over nickel sulfide (NiS) produced three-dimensional flower-like structures which showed electrochemical properties and charge storage performance. Sangabathula et al. introduced a binder-free technique to incorporate GQDs inside the molybdenum nickel sulfide (MNS) through a one-step hydrothermal process. Here, flower-like morphology in GQD/MNS produced more active edges. The asymmetric capacitor was constructed by using GQD/MNS as the positive electrode, and rGO as the negative electrode, delivering a good coulombic efficiency and sufficient capacitance retention. An ultra-high specific capacitance of 2622 F g^−1^ was measured at 1 A g^−1^ current density for the asymmetric capacitor [184,185]. Apart from the electrode materials, GQDs have also been utilized as electrolytes for solid state supercapacitors [184]. Huang’s group proposed that, due to the presence of sufficient numbers of oxygen-containing functional groups, they can serve as electrolytes both in the solution and solid phase. It was found that ion-donating ability and ionic conductivity of GQDs could be improved simply by neutralizing the acidic surface groups. Such neutralized GQDs, as the electrolytes, greatly improved the capacitive performance. However, more research is required to gain detailed insights on such activity of GQDs. The extraordinary performance of GQD-based supercapacitor electrodes has been tabulated in Table 3.

## 5. Conclusions and Perspectives

In summary, the manuscript addresses the importance of GQDs and GQD-based composites in the field of energy storage applications. Recent reports on the interesting properties of GQDs suggest that they have the great potential to appear as a sustainable and efficient material for electrodes. Certain properties of GQDs, such as highly conductive networks, high specific surface area, and presence of different surface functional groups, are helpful for energy-related applications. These properties introduce pseudocapacitive behavior along with reduced charge-transfer resistance, cyclic stability, and enhanced specific capacitance to GQD-based electrodes. Thus, recent studies on the GQD-based energy-related applications have become a pivotal attention for batteries and supercapacitor studies. Besides, many knowledge gaps still persist, and more studies are still required in order to establish full structural advantages of GQDs. For example, (1) effect of doped heteroatoms on the surface properties GQDs are not still clear. A detailed understanding is required on how the doped heteroatoms affect the specific surface area, crystallinity, and ion-transportation ability in the electrode materials prepared with GQDs and their composites. (2) To date, several experimental reports have been made on the fundamental properties of GQDs. However, the effect of defect and edge states, crystallinity, and heteroatoms toward the electrochemical performance of GQDs with appropriate mechanisms still remain unclear. (3) It is essential to synthesize GQDs at industrial scale with high crystallinity and good yield. (4) Development of stable composite electrode materials are still urgent. The surface functional groups, in this regard, will have a crucial role to produce composite-based electrodes with long-term cyclic stability. A deep mechanistic study on the interaction of GQDs with other materials is therefore highly needed. Thus, a lot of room is still available to explore the field of GQD-based energy storage research. In summary, GQDs have provided a completely new dimension to the batteries and supercapacitors research. It is believed that, with the help of more research and innovations, as mentioned above, high-performance sustainable electrochemical devices for energy storage can be developed in the near future.

## Figures and Tables

**Table 1:** Specific surface area and series resistance (Rs) or the charge transfer resistance (Rct) changes due to incorporation of GQDs in the composite electrode materials.

Electrode Material	Specific Surface Area of the Pristine Material (m^2^g^−1^)	Series Resistance (R_s_) and Charge Transfer Resistance (R_ct_) of the Pristine Material (Ω)	Specific Surface Area of GQDs-Based Electrode Material (m^2^g^−1^)	Series Resistance (R_s_) and Charge Transfer Resistance (R_ct_) of GQDs-Based Material (Ω)	Ref.
N-GQD/cMOF-5	899.8	3.25 (R_s_)	704.1	1.91 (R_s_)	[108]
Activated GQDs on glassy carbon	1289	-	1502	-	[110]
Activated carbon nanofiber/GQDs	140	1.84 (R_ct_)	2032	0.37 (R_ct_)	[112]
Ultra-microporous carbon/GQDs	69	-	1780	-	[113]
Three-dimensional graphene/GQDs	192	-	292	-	[116]
Activated carbon/GQDs	1600	3.2 (R_ct_)	2829	0.1 (R_ct_)	[117]
MnO_2_ nanosheets/GQDs	77	-	108	-	[118]
N-GQDs/GH/CF	3.22	-	13.48	-	[119]
N-GQD@cZIF-8/CNT	840	0.97 (R_ct_)	520	0.71 (R_ct_)	[120]
GQD/CNT/carbon cloth	95	0.79 (R_s_)	~400	0.71 (R_s_)	[126]

**Table 2:** Electrochemical performances of the GQD-based battery electrodes.

Electrode Materials	Type of Batteries	Capacity (mAh/g)	Retention (%)	Ref.
GQDs-VO2	SIB	306@1/3C	88% after 1500 cycles	[127]
GQDs derived p-doped carbon sheets	SIB	328@0.1 A/g	104% after 5000 cycles	[128]
Nitrogen doped GQDs	LiS	1330.0@0.5C	99.9% after 500 cycles	[129]
GQDs decorated S	Lis	1200.0@0.5C	75% after 100 cycles	[130]
GQDs-MoS2	LiB	1099.0@0.1 A/g	87% after 80 cycles	[131]
GQD-NiO	LiB	1081.0@0.1C	120% after 250 cycles	[132]
GQDs-TiO2-x	LiB	227.0@0.1C	160.1% after 500 cycles	[133]
GQDs-VO2	LiB	421.0@1/3C	94% after 1500 cycles	[134]
GQDs-B	LiB	859.0@0.05 A/g	95.7% after 500 cycles	[127]

**Table 3:** Electrochemical performances of the GQD-based supercapacitor electrodes.

Precursors Used for GQD Synthesis	GQD Synthesis Process	Electrode Materials	Specific Capacitance	Cycling Stability	Energy Density (Wh kg^−1^)	Power Density (W kg^−1^)	Ref.
Citric acid and thiourea	Heating at 160 °C for 6 h in a Teflon-lined autoclave.	N,S-GQD/PANI	645 F g^−1^	90% (1000 cycles)	17.25	500	[106]
GO powder	Heating at 200 °C for 24 h in a Teflon-lined autoclave.	GQD/NiCo_2_O_4_	1242 F g^−1^	99% (4000 cycles)	38	800	[107]
Pyrene	Nitration followed by ultrasonication and heating at 200 °C for 12 h in a Teflon-lined autoclave.	N-GQD/cMOF	780 F g^−1^	94.1% (5000 cycles)	14.4	400.6	[108]
Graphene sheets	Heating at 800 °C for 2 h in an inert atmosphere.	Activated GQDs	236 F g^−1^	-	-	-	[110]
Urea and citric acid	Heating at 180 °C for 3 h in a Teflon-lined autoclave.	N-GQD/halloysite nanotubes	335 F g^−1^	95% (3000 cycles)	-	-	[115]
Carbon rod	Electrochemical method	GQD/three-dimensional graphene	268 F g^−1^	90% (5000 cycles)	-	-	[116]
Bituminous coal powder	Chemical oxidation	GQD/activated carbon	388 F g^−1^	100% (10,000 cycles)	13.47	125	[117]
Pyrene	Molecular fusion	N-GQD/graphene hydrogel/carbon fibers	93.7 F cm^−3^	87.9% (5000 cycles)	3.6 mWh cm^−3^	35.6 mWcm^−3^	[119]
Pyrene	Hydrothermal molecular fusion.	GQD/TiO_2_ nanotube	595 F g^−1^	90% (10,000 cycles)	21.8	0.25 kW kg^−1^	[124]
-	Carboxyl-functionalized GQDs (commercially procured)	NiO/Co_3_O_4_/GQDs	1361 F g^−1^	84.3% (10,000 cycles)	38.44	750	[130]
GO powder (prepared form graphite)	H_2_O_2_ treatment at 90 °C.	GQD-PANI	1044 F g^−1^	80.1% (3000 cycles)	117.45	448.8	[167]
Carbon fibers	Exfoliation by mixed acids	PANI/GQD-rGO/CFC	1036 F g^−1^	97.7% (10,000 cycles)	34.2	424.4	[169]
Citric acid	Heating at 200 °C	GQD/PPy	284.01 F g^−1^	86% (5000 cycles)	81.79	18.17 kW kg^−1^	[171]
GO solution	H_2_O_2_ treatment at 90 °C for 12 h.	GQD/MnO_2_	1170 F g^−1^	92.7% (10,000 cycles)	118	923	[174]
Pyrene	HNO_3_ treatment and heating up to 200 °C for 10 h.	GQD/V_2_O_5_	572 F g^−1^	92% (10,000 cycles)	20.62	14.86 kW kg^−1^	[176]
Citric acid	Heating at 200 °C for 30 min.	CuMnO_2_/GQD	153.2 F g^−1^	86.7% (5000 cycles)	47.9	1108.1	[181]
Citric acid	Heating at 200 °C for 40 min.	MnCo_2_O_4,5_/GQD	1625 F g^−1^	77% (5000 cycles)	46	66	[182]
Citric acid and tryptophan	Pyrolysis	NiCo_2_S_4_/Trp-GQD	1453.1 F g^−1^	~94.8% (5000 cycles)	157.1	800	[183]
Urea and citric acid	Hydrothermal	GQD/MNS	2622 F g^−1^	98% (10,000 cycles)	38.9	416.6	[184]

## Data Availability

Data available in a publicly accessible repository.

## References

[1] Salanne M., Rotenberg B., Naoi K., Kaneko K., Taberna P.L., Grey C.P., Dunn B., Simon P. (2016). Efficient storage mechanisms for building better supercapacitors. Nat. Energy.

[2] Zuo W., Li R., Zhou C., Li Y., Xia J., Liu J. (2017). Battery-Supercapacitor Hybrid Devices: Recent Progress and Future Prospects. Adv. Sci..

[3] Kumar K.S., Choudhary N., Jung Y., Thomas J. (2018). Recent Advances in Two-Dimensional Nanomaterials for Supercapacitor Electrode Applications. ACS Energy Lett..

[4] Choudhary N., Li C., Moore J., Nagaiah N., Zhai L., Jung Y., Thomas J. (2017). Asymmetric Supercapacitor Electrodes and Devices. Adv. Mater..

[5] Shao Y., El-Kady M.F., Sun J., Li Y., Zhang Q., Zhu M., Wang H., Dunn B., Kaner R.B. (2018). Design and Mechanisms of Asymmetric Supercapacitors. Chem. Rev..

[6] Gao Y., Zhao L. (2022). Review on recent advances in nanostructured transition-metal-sulfide-based electrode materials for cathode materials of asymmetric supercapacitors. Chem. Eng. J..

[7] Ansari M.Z., Ansari S.A., Kim S.H. (2022). Fundamentals and recent progress of Sn-based electrode materials for supercapacitors: A comprehensive review. J. Energy Storage.

[8] Parveen N., Ansari S.A., Ansari M.Z., Ansari M.O. (2022). Manganese oxide as an effective electrode material for energy storage: A review. Environ. Chem. Lett..

[9] Zhang Y., Li L., Su H., Huang W., Dong X. (2015). Binary metal oxide: Advanced energy storage materials in supercapacitors. J. Mater. Chem. A Mater..

[10] Zhou Y., Qi H., Yang J., Bo Z., Huang F., Islam M.S., Lu X., Dai L., Amal R., Wang C.H. (2021). Two-birds-one-stone: Multifunctional supercapacitors beyond traditional energy storage. Energy Environ. Sci..

[11] Ansari S.A., Parveen N., Al-Othoum M.A.S., Ansari M.O. (2021). Effect of Washing on the Electrochemical Performance of a Three-Dimensional Current Collector for Energy Storage Applications. Nanomaterials.

[12] Chen T., Dai L. (2013). Carbon nanomaterials for high-performance supercapacitors. Mater. Today.

[13] Hu Y., Cheng H., Zhao F., Chen N., Jiang L., Feng Z., Qu L. (2014). All-in-one graphene fiber supercapacitor. Nanoscale.

[14] Han J., Zhang L.L., Lee S., Oh J., Lee K.S., Potts J.R., Ji J., Zhao X., Ruoff R.S., Park S. (2013). Generation of B-doped graphene nanoplatelets using a solution process and their supercapacitor applications. ACS Nano.

[15] Novoselov K.S., Fal’Ko V.I., Colombo L., Gellert P.R., Schwab M.G., Kim K. (2012). A roadmap for graphene. Nature.

[16] Vicarelli L., Heerema S.J., Dekker C., Zandbergen H.W. (2015). Controlling defects in graphene for optimizing the electrical properties of graphene nanodevices. ACS Nano.

[17] Worsley M.A., Kucheyev S.O., Mason H.E., Merrill M.D., Mayer B.P., Lewicki J., Valdez C.A., Suss M.E., Stadermann M., Pauzauskie P.J. (2012). Mechanically robust 3D graphene macroassembly with high surface area. Chem. Commun..

[18] Zhu J., Yang D., Yin Z., Yan Q., Zhang H. (2014). Graphene and graphene-based materials for energy storage applications. Small.

[19] Dong Z., Jiang C., Cheng H., Zhao Y., Shi G., Jiang L., Qu L. (2012). Facile fabrication of light, flexible and multifunctional graphene fibers. Adv. Mater..

[20] Salihoglu O., Kakenov N., Balci O., Balci S., Kocabas C. (2018). Graphene-Quantum Dot Hybrid Optoelectronics at Visible Wavelengths. ACS Photonics..

[21] Gupta V., Chaudhary N., Srivastava R., Sharma G.D., Bhardwaj R., Chand S. (2011). Luminscent graphene quantum dots for organic photovoltaic devices. J Am. Chem. Soc..

[22] Shen J., Zhu Y., Chen C., Yang X., Li C. (2011). Facile preparation and upconversion luminescence of graphene quantum dots. Chem. Commun..

[23] Van Tam T., Kang S.G., Kim M.H., Lee S.G., Hur S.H., Chung J.S., Choi W.M. (2019). Novel Graphene Hydrogel/B-Doped Graphene Quantum Dots Composites as Trifunctional Electrocatalysts for Zn-Air Batteries and Overall Water Splitting. Adv. Energy Mater..

[24] Diao S., Zhang X., Shao Z., Ding K., Jie J., Zhang X. (2017). 12.35% efficient graphene quantum dots/silicon heterojunction solar cells using graphene transparent electrode. Nano Energy.

[25] Shaari N., Kamarudin S.K., Bahru R. (2021). Carbon and graphene quantum dots in fuel cell application: An overview. Int. J. Energy Res..

[26] Khose R.V., Chakraborty G., Bondarde M.P., Wadekar P.H., Ray A.K., Some S. (2021). Red-fluorescent graphene quantum dots from guava leaf as a turn-off probe for sensing aqueous Hg(II). New J. Chem..

[27] Sheely A., Gifford B., Tretiak S., Bishop A. (2021). Tunable Optical Features of Graphene Quantum Dots from Edge Functionalization. J. Phys. Chem. C.

[28] Li Y., Shu H., Wang S., Wang J. (2015). Electronic and optical properties of graphene quantum dots: The role of many-body effects. J. Phys. Chem. C.

[29] Kaur M., Kaur M., Sharma V.K. (2018). Nitrogen-doped graphene and graphene quantum dots: A review onsynthesis and applications in energy, sensors and environment. Adv. Colloid. Interface Sci..

[30] Daugherty M.C., Gu S., Aaron D.S., Kelly R.E., Gandomi Y.A., Hsieh C. (2020). Graphene quantum dot-decorated carbon electrodes for energy storage in vanadium redox flow batteries. Nanoscale.

[31] Zhu J., Wang L., Gan X., Tang T., Qin F., Luo W., Li Q., Guo N., Zhang S., Jia D. (2022). Graphene quantum dot inlaid carbon nanofibers: Revealing the edge activity for ultrahigh rate pseudocapacitive energy storage. Energy Storage Mater..

[32] El-Kady M.F., Shao Y., Kaner R.B. (2016). Graphene for batteries, supercapacitors and beyond. Nat. Rev. Mater..

[33] Pan D., Zhang J., Li Z., Wu M. (2010). Hydrothermal route for cutting graphene sheets into blue-luminescent graphene quantum dots. Adv. Mater..

[34] Pan D., Guo L., Zhang J., Xi C., Xue Q., Huang H., Li J., Zhang Z., Yu W., Chen Z. (2012). Cutting sp^2^ clusters in graphene sheets into colloidal graphene quantum dots with strong green fluorescence. J. Mater. Chem..

[35] Sun Y., Wang S., Li C., Luo P., Tao L., Wei Y., Shi G. (2013). Large scale preparation of graphene quantum dots from graphite with tunable fluorescence properties. Phys. Chem. Chem. Phys..

[36] Zhao Y., Wu X., Sun S., Ma L., Zhang L., Lin H. (2017). A facile and high-efficient approach to yellow emissive graphene quantum dots from graphene oxide. Carbon.

[37] Zhu X., Xiao X., Zuo X., Liang Y., Nan J. (2014). Hydrothermal preparation of photoluminescent graphene quantum dots characterized excitation-independent emission and its application as a bioimaging reagent. Part. Part. Syst. Charact..

[38] Kellici S., Acord J., Power N.P., Morgan D.J., Coppo P., Heil T., Saha B. (2017). Rapid synthesis of graphene quantum dots using a continuous hydrothermal flow synthesis approach. RSC Adv..

[39] Zhu S., Zhang J., Liu X., Li B., Wang X., Tang S., Meng Q., Li Y., Shi C., Hu R. (2012). Graphene quantum dots with controllable surface oxidation, tunable fluorescence and up-conversion emission. RSC Adv..

[40] Fang B.Y., Li C., Song Y.Y., Tan F., Cao Y.C., di Zhao Y. (2018). Nitrogen-doped graphene quantum dot for direct fluorescence detection of Al^3+^ in aqueous media and living cells. Biosens. Bioelectron..

[41] Tian R., Zhong S., Wu J., Jiang W., Shen Y., Jiang W., Wang T. (2016). Solvothermal method to prepare graphene quantum dots by hydrogen peroxide. Opt. Mater..

[42] Shin Y., Park J., Hyun D., Yang J., Lee J.H., Kim J.H., Lee H. (2015). Acid-free and oxone oxidant-assisted solvothermal synthesis of graphene quantum dots using various natural carbon materials as resources. Nanoscale.

[43] Shinde D.B., Pillai V.K. (2012). Electrochemical preparation of luminescent graphene quantum dots from multiwalled carbon nanotubes. Chem. A Eur. J..

[44] Tan X., Li Y., Li X., Zhou S., Fan L., Yang S. (2015). Electrochemical synthesis of small-sized red fluorescent graphene quantum dots as a bioimaging platform. Chem. Commun..

[45] Li Y., Li S., Wang Y., Wang J., Liu H., Liu X., Wang L., Liu X., Xue W., Ma N. (2017). Electrochemical synthesis of phosphorus-doped graphene quantum dots for free radical scavenging. Phys. Chem. Chem. Phys..

[46] Kalita H., Palaparthy V.S., Baghini M.S., Aslam M. (2020). Electrochemical synthesis of graphene quantum dots from graphene oxide at room temperature and its soil moisture sensing properties. Carbon.

[47] Lu L., Zhu Y., Shi C., Pei Y.T. (2016). Large-scale synthesis of defect-selective graphene quantum dots by ultrasonic-assisted liquid-phase exfoliation. Carbon.

[48] Zhuo S., Shao M., Lee S.T. (2012). Upconversion and downconversion fluorescent graphene quantum dots: Ultrasonic preparation and photocatalysis. ACS Nano.

[49] Zhang Y., Li K., Ren S., Dang Y., Liu G., Zhang R., Zhang K., Long X., Jia K. (2019). Coal-Derived Graphene Quantum Dots Produced by Ultrasonic Physical Tailoring and Their Capacity for Cu(II) Detection. ACS Sustain. Chem Eng..

[50] Kang S., Ryu J.H., Lee B., Jung K.H., Shim K.B., Han H., Kim K.M. (2019). Laser wavelength modulated pulsed laser ablation for selective and efficient production of graphene quantum dots. RSC Adv..

[51] Qin Y., Cheng Y., Jiang L., Jin X., Li M., Luo X., Liao G., Wei T., Li Q. (2015). Top-down strategy toward versatile graphene quantum dots for organic/inorganic hybrid solar cells. ACS Sustain. Chem. Eng..

[52] Zhang A., Chen T., Song S., Yang W., Gooding J.J., Liu J. (2021). Ultrafast generation of highly crystalline graphene quantum dots from graphite paper via laser writing. J Colloid Interface Sci..

[53] Calabro R.L., Yang D.S., Kim D.Y. (2018). Liquid-phase laser ablation synthesis of graphene quantum dots from carbon nano-onions: Comparison with chemical oxidation. J. Colloid Interface Sci..

[54] Kang S., Kim K.M., Jung K., Son Y., Mhin S., Ryu J.H., Shim K.B., Lee B., Han H.S., Song T. (2019). Graphene Oxide Quantum Dots Derived from Coal for Bioimaging: Facile and Green Approach. Sci. Rep..

[55] Li L.L., Ji J., Fei R., Wang C.Z., Lu Q., Zhang J.R., Jiang L.P., Zhu J.J. (2012). A facile microwave avenue to electrochemiluminescent two-color graphene quantum dots. Adv. Funct. Mater..

[56] Sun H., Ji H., Ju E., Guan Y., Ren J., Qu X. (2015). Synthesis of fluorinated and nonfluorinated graphene quantum dots through a new top-down strategy for long-time cellular imaging. Chem. A Eur. J..

[57] Gu S., Hsieh C.t., Chiang Y.M., Tzou D.Y., Chen Y.F., Gandomi Y.A. (2018). Optimization of graphene quantum dots by chemical exfoliation from graphite powders and carbon nanotubes. Mater. Chem. Phys..

[58] Wei S., Zhang R., Liu Y., Ding H., Zhang Y.L. (2016). Graphene quantum dots prepared from chemical exfoliation of multiwall carbon nanotubes: An efficient photocatalyst promoter. Catal. Commun..

[59] Neubeck S., Ponomarenko L.A., Freitag F., Giesbers A.J.M., Zeitler U., Morozov S.v., Blake P., Geim A.K., Novoselov K.S. (2010). From one electron to one hole: Quasiparticle counting in graphene quantum dots determined by electrochemical and plasma etching. Small.

[60] Ponomarenko L.A., Schedin F., Katsnelson M.I., Yang R., Hill E.W., Novoselov K.S., Geim A.K. (2008). Chaotic dirac billiard in graphene quantum dots. Science.

[61] Jeon S.J., Kang T.W., Ju J.M., Kim M.J., Park J.H., Raza F., Han J., Lee H.R., Kim J.H. (2016). Modulating the Photocatalytic Activity of Graphene Quantum Dots via Atomic Tailoring for Highly Enhanced Photocatalysis under Visible Light. Adv. Funct. Mater..

[62] Li W., Li M., Liu Y., Pan D., Li Z., Wang L., Wu M. (2018). Three Minute Ultrarapid Microwave-Assisted Synthesis of Bright Fluorescent Graphene Quantum Dots for Live Cell Staining and White LEDs. ACS Appl. Nano Mater..

[63] Huang W., Li X., Sun X., Ding X., Feng Y., Tang Y., Zhou P., Wang L., Zhang Q. (2021). Photoluminescence of graphene quantum dots enhanced by microwave post-treatment. Chem. Eng. J..

[64] Umrao S., Jang M.H., Oh J.H., Kim G., Sahoo S., Cho Y.H., Srivastva A., Oh I.K. (2015). Microwave bottom-up route for size-tunable and switchable photoluminescent graphene quantum dots using acetylacetone: New platform for enzyme-free detection of hydrogen peroxide. Carbon.

[65] Qiu H., Sun X., An S., Lan D., Cui J., Zhang Y., He W. (2020). Microwave synthesis of histidine-functionalized graphene quantum dots/Ni-Co LDH with flower ball structure for supercapacitor. J. Colloid Interface Sci..

[66] Mahesh S., Lekshmi C.L., Renuka K.D., Joseph K. (2016). Simple and Cost-Effective Synthesis of Fluorescent Graphene Quantum Dots from Honey: Application as Stable Security Ink and White-Light Emission. Part. Part. Syst. Charact..

[67] Abbas A., Tabish T.A., Bull S.J., Lim T.M., Phan A.N. (2020). High yield synthesis of graphene quantum dots from biomass waste as a highly selective probe for Fe^3+^ sensing. Sci. Rep..

[68] Zhang C., Cui Y., Song L., Liu X., Hu Z. (2016). Microwave assisted one-pot synthesis of graphene quantum dots as highly sensitive fluorescent probes for detection of iron ions and pH value. Talanta.

[69] Ganganboina A.B., Chowdhury A.D., Doong R.A. (2018). N-Doped Graphene Quantum Dots-Decorated V_2_O_5_ Nanosheet for Fluorescence Turn Off-On Detection of Cysteine. ACS Appl. Mater. Interfaces.

[70] Gu J., Zhang X., Pang A., Yang J. (2016). Facile synthesis and photoluminescence characteristics of blue-emitting nitrogen-doped graphene quantum dots. Nanotechnology.

[71] Ogi T., Iwasaki H., Aishima K., Iskandar F., Wang W.N., Takimiya K., Okuyama K. (2014). Transient nature of graphene quantum dot formation via a hydrothermal reaction. RSC Adv..

[72] Lin L., Rong M., Lu S., Song X., Zhong Y., Yan J., Wang Y., Chen X. (2015). A facile synthesis of highly luminescent nitrogen-doped graphene quantum dots for the detection of 2,4,6-trinitrophenol in aqueous solution. Nanoscale.

[73] Ju J., Zhang R., He S., Chen W. (2014). Nitrogen-doped graphene quantum dots-based fluorescent probe for the sensitive turn-on detection of glutathione and its cellular imaging. RSC Adv..

[74] Safardoust-Hojaghan H., Amiri O., Hassanpour M., Panahi-Kalamuei M., Moayedi H., Salavati-Niasari M. (2019). S, N co-doped graphene quantum dots-induced ascorbic acid fluorescent sensor: Design, characterization and performance. Food Chem..

[75] Wang L., Wang Y., Xu T., Liao H., Yao C., Liu Y., Li Z., Chen Z., Pan D., Sun L. (2014). Gram-scale synthesis of single-crystalline graphene quantum dots with superior optical properties. Nat. Commun..

[76] Guo Z., Cai B., Cao Q., Su Y., Li M., Hu J., Yang Z., Zhang Y. (2017). Facile synthesis of amine-functionalized graphene quantum dots with highly pH-sensitive photoluminescence. Fuller. Nanotub. Carbon Nanostruct..

[77] Tang L., Ji R., Li X., Teng K.S., Lau S.P. (2013). Size-dependent structural and optical characteristics of glucose-derived graphene quantum dots. Part. Part. Syst. Charact..

[78] Liu R., Wu D., Feng X., Müllen K. (2011). Bottom-up fabrication of photoluminescent graphene quantum dots with uniform morphology. J. Am. Chem. Soc..

[79] Do S., Kwon W., Rhee S.W. (2014). Soft-template synthesis of nitrogen-doped carbon nanodots: Tunable visible-light photoluminescence and phosphor-based light-emitting diodes. J. Mater. Chem C Mater..

[80] Gao S., Tang L., Xiang J., Ji R., Lai S.K., Yuan S., Lau S.P. (2017). Facile preparation of sulphur-doped graphene quantum dots for ultra-high performance ultraviolet photodetectors. New J. Chem..

[81] Yan X., Cui X., Li L.S. (2010). Synthesis of large, stable colloidal graphene quantum dots with tunable size. J. Am. Chem. Soc..

[82] Yan X., Li B., Cui X., Wei Q., Tajima K., Li L.S. (2011). Independent tuning of the band gap and redox potential of graphene quantum dots. J. Phys. Chem. Lett..

[83] Shen J., Zhu Y., Yang X., Li C. (2021). Graphene quantum dots: Emergent nanolights for bioimaging, sensors, catalysis and photovoltaic devices. Chem. Commun..

[84] Zhang P., Hu Q., Yang X., Hou X., Mi J., Liu L., Dong M. (2018). Size effect of oxygen reduction reaction on nitrogen-doped graphene quantum dots. RSC Adv..

[85] Bian S., Shen C., Hua H., Zhou L., Zhu H., Xi F., Liu J., Dong X. (2016). One-pot synthesis of sulfur-doped graphene quantum dots as a novel fluorescent probe for highly selective and sensitive detection of lead(II). RSC Adv..

[86] Yang G., Wu C., Luo X., Liu X., Gao Y., Wu P., Cai C., Saavedra S.S. (2018). Exploring the Emissive States of Heteroatom-Doped Graphene Quantum Dots. J. Phys. Chem. C.

[87] Zhang L., Zhang Z.Y., Liang R.P., Li Y.H., Qiu J.D. (2014). Boron-doped graphene quantum dots for selective glucose sensing based on the “abnormal” aggregation-induced photoluminescence enhancement. Anal. Chem..

[88] Lee S.H., Kim D.Y., Lee J., Lee S.B., Han H., Kim Y.Y., Mun S.C., Im S.H., Kim T.H., Park O.O. (2019). Synthesis of Single-Crystalline Hexagonal Graphene Quantum Dots from Solution Chemistry. Nano Lett..

[89] Qu D., Zheng M., Zhang L., Zhao H., Xie Z., Jing X., Haddad R.E., Fan H., Sun Z. (2014). Formation mechanism and optimization of highly luminescent N-doped graphene quantum dots. Sci. Rep..

[90] Xu Y., Bai H., Lu G., Li C., Shi G. (2008). Flexible graphene films via the filtration of water-soluble noncovalent functionalized graphene sheets. J. Am. Chem. Soc..

[91] Katsnelson M.I. (2007). Graphene: Carbon in two dimensions. Mater. Today.

[92] Ma L., Wang J., Ding F. (2013). Recent progress and challenges in graphene nanoribbon synthesis. ChemPhysChem.

[93] McIver J.W., Schulte B., Stein F.U., Matsuyama T., Jotzu G., Meier G., Cavalleri A. (2020). Light-induced anomalous Hall effect in graphene. Nat. Phys..

[94] Adjizian J.J., Briddon P., Humbert B., Duvail J.L., Wagner P., Adda C., Ewels C. (2014). Dirac Cones in two-dimensional conjugated polymer networks. Nat. Commun..

[95] Morozov S.v., Novoselov K.S., Katsnelson M.I., Schedin F., Elias D.C., Jaszczak J.A., Geim A.K. (2008). Giant intrinsic carrier mobilities in graphene and its bilayer. Phys. Rev. Lett..

[96] Bolotin K.I., Sikes K.J., Jiang Z., Klima M., Fudenberg G., Hone J., Kim P., Stormer H.L. (2008). Ultrahigh electron mobility in suspended graphene. Solid State Commun..

[97] Sang M., Shin J., Kim K., Yu K.J. (2019). Electronic and thermal properties of graphene and recent advances in graphene based electronics applications. Nanomaterials.

[98] Gosling J.H., Makarovsky O., Wang F., Cottam N.D., Greenaway M.T., Patanè A., Wildman R.D., Tuck C.J., Turyanska L., Fromhold T.M. (2021). Universal mobility characteristics of graphene originating from charge scattering by ionised impurities. Commun. Phys..

[99] Jia Y., Gong X., Peng P., Wang Z., Tian Z., Ren L., Fu Y., Zhang H. (2016). Toward High Carrier Mobility and Low Contact Resistance: Laser Cleaning of PMMA Residues on Graphene Surfaces. Nanomicro Lett..

[100] Choi M.S., Nipane A., Kim B.S.Y., Ziffer M.E., Datta I., Borah A., Jung Y., Kim B., Rhodes D., Jindal A. (2021). High carrier mobility in graphene doped using a monolayer of tungsten oxyselenide. Nat. Electron..

[101] Schwartz M.A. (2009). Cell biology: The force is with us. Science.

[102] Katsnelson M.I., Novoselov K.S., Geim A.K. (2006). Chiral tunnelling and the Klein paradox in graphene. Nat. Phys..

[103] Gao L., Theuns T. (2007). Lighting the universe with filaments. Science.

[104] Peng J., Gao W., Gupta B.K., Liu Z., Romero-Aburto R., Ge L., Song L., Alemany L.B., Zhan X., Gao G. (2021). Graphene quantum dots derived from carbon fibers. Nano Lett..

[105] Jin S.H., Kim D.H., Jun G.H., Hong S.H., Jeon S. (2013). Tuning the photoluminescence of graphene quantum dots through the charge transfer effect of functional groups. ACS Nano.

[106] Kuzhandaivel H., Manickam S., Balasingam S.K., Franklin M.C., Kim H.J., Nallathambi K.S. (2021). Sulfur and nitrogen-doped graphene quantum dots/PANI nanocomposites for supercapacitors. New J. Chem..

[107] Luo J., Wang J., Liu S., Wu W., Jia T., Yang Z., Mu S., Huang Y. (2019). Graphene quantum dots encapsulated tremella-like NiCo_2_O_4_ for advanced asymmetric supercapacitors. Carbon.

[108] Li Z., Bu F., Wei J., Yao W., Wang L., Chen Z., Pan D., Wu M. (2018). Boosting the energy storage densities of supercapacitors by incorporating N-doped graphene quantum dots into cubic porous carbon. Nanoscale.

[109] Abbas A., Mariana L.T., Phan A.N. (2018). Biomass-waste derived graphene quantum dots and their applications. Carbon.

[110] Hassan M., Haque E., Reddy K.R., Minett A.I., Chen J., Gomes V.G. (2014). Edge-enriched graphene quantum dots for enhanced photo-luminescence and supercapacitance. Nanoscale.

[111] Zhang S., Sui L., Dong H., He W., Dong L., Yu L. (2018). High-Performance Supercapacitor of Graphene Quantum Dots with Uniform Sizes. ACS Appl. Mater. Interfaces.

[112] Zhao J., Zhu J., Li Y., Wang L., Dong Y., Jiang Z., Fan C., Cao Y., Sheng R., Liu A. (2020). Graphene Quantum Dot Reinforced Electrospun Carbon Nanofiber Fabrics with High Surface Area for Ultrahigh Rate Supercapacitors. ACS Appl. Mater. Interfaces.

[113] Zhang S., Zhu J., Qing Y., Wang L., Zhao J., Li J., Tian W., Jia D., Fan Z. (2018). Ultramicroporous Carbons Puzzled by Graphene Quantum Dots: Integrated High Gravimetric, Volumetric, and Areal Capacitances for Supercapacitors. Adv. Funct. Mater..

[114] Hu Y., Zhao Y., Lu G., Chen N., Zhang Z., Li H., Shao H., Qu L. (2013). Graphene quantum dots-carbon nanotube hybrid arrays for supercapacitors. Nanotechnology.

[115] Ganganboina A.B., Doong R.A. (2020). Nitrogen doped graphene quantum dot-decorated earth-abundant nanotubes for enhanced capacitive deionization. Environ. Sci. Nano.

[116] Chen Q., Hu Y., Hu C., Cheng H., Zhang Z., Shao H., Qu L. (2014). Graphene quantum dots-three-dimensional graphene composites for high-performance supercapacitors. Phys. Chem. Chem. Phys..

[117] Qing Y., Jiang Y., Lin H., Wang L., Liu A., Cao Y., Sheng R., Guo Y., Fan C., Zhang S. (2019). Boosting the supercapacitor performance of activated carbon by constructing overall conductive networks using graphene quantum dots. J. Mater. Chem. A Mater..

[118] Zhu H., Li L., Shi M., Xiao P., Liu Y., Yan X. (2022). Coupling of graphene quantum dots with MnO_2_ nanosheets for boosting capacitive storage in ionic liquid electrolyte. Chem. Eng. J..

[119] Geethalakshmi K.R., Ng T.Y., Crespo-Otero R. (2016). Tunable optical properties of OH-functionalised graphene quantum dots. J. Mater. Chem. C Mater..

[120] Li Z., Liu X., Wang L., Bu F., Wei J., Pan D., Wu M. (2018). Hierarchical 3D All-Carbon Composite Structure Modified with N-Doped Graphene Quantum Dots for High-Performance Flexible Supercapacitors. Small.

[121] Li J., Dong Y., Zhu J., Wang L., Tian W., Zhao J., Lin H., Zhang S., Cao Y., Song H. (2020). N co-doped carbon nanosheets derived from graphene quantum dots: Improving the pseudocapacitive performance by efficient trapping nitrogen. Appl. Surf. Sci..

[122] Sim Y., Kim S.J., Janani G., Chae Y., Surendran S., Kim H., Yoo S., Seok D.C., Jung Y.H., Jeon C. (2020). The synergistic effect of nitrogen and fluorine co-doping in graphene quantum dot catalysts for full water splitting and supercapacitor. Appl. Surf. Sci..

[123] Hsiao Y.J., Lin L.Y. (2020). Enhanced Surface Area, Graphene Quantum Dots, and Functional Groups for the Simple Acid-Treated Carbon Fiber Electrode of Flexible Fiber-Type Solid-State Supercapacitors without Active Materials. ACS Sustain. Chem. Eng..

[124] Li Z., Qin P., Wang L., Yang C., Li Y., Chen Z., Pan D., Wu M. (2016). Amine-enriched Graphene Quantum Dots for High-pseudocapacitance Supercapacitors. Electrochim. Acta..

[125] Liu W., Yan X., Chen J., Feng Y., Xue Q. (2013). Novel and high-performance asymmetric micro-supercapacitors based on graphene quantum dots and polyaniline nanofibers. Nanoscale.

[126] Li Z., Li Y., Wang L., Cao L., Liu X., Chen Z., Pan D., Wu M. (2017). Assembling nitrogen and oxygen co-doped graphene quantum dots onto hierarchical carbon networks for all-solid-state flexible supercapacitors. Electrochim Acta..

[127] Saroja A.P.V.K., Garapati M.S., Devi R.S., Kamaraj M., Ramaprabhu S. (2020). Facile synthesis of heteroatom doped and undoped graphene quantum dots as active materials for reversible lithium and sodium ions storage. Appl. Surf. Sci..

[128] Zhang Q., Sun C., Fan L., Zhang N., Sun K. (2019). Iron fluoride vertical nanosheets array modified with graphene quantum dots as long-life cathode for lithium ion batteries. Chem. Eng. J..

[129] Zhang W., Xu T., Liu Z., Wu N.-L., Wei M. (2018). Hierarchical TiO2−x imbedded with graphene quantum dots for high-performance lithium storage. Chem. Commun..

[130] Yin X., Zhi C., Sun W., Lv L.-P., Wang Y. (2019). Multilayer NiO@Co3O4@graphene quantum dots hollow spheres for high-performance lithium-ion batteries and supercapacitors. J. Mater. Chem. A.

[131] Guo J., Zhu H., Sun Y., Tang L., Zhang X. (2016). Boosting the lithium storage performance of MoS2 with graphene quantum dots. J. Mater. Chem. A.

[132] Park J., Moon J., Kim C., Kang J.H., Lim E., Park J., Lee K.J., Yu S.-H., Seo J.-H., Lee J. (2016). Graphene quantum dots: Structural integrity and oxygen functional groups for high sulfur/sulfide utilization in lithium sulfur batteries. NPG Asia Mater..

[133] Pang Y., Wei J., Wang Y., Xia Y. (2018). Synergetic Protective Effect of the Ultralight MWCNTs/NCQDs Modified Separator for Highly Stable Lithium–Sulfur Batteries. Adv. Energy Mater..

[134] Hong W., Zhang Y., Yang L., Tian Y., Ge P., Hu J., Wei W., Zou G., Hou H., Ji X. (2019). Carbon quantum dot micelles tailored hollow carbon anode for fast potassium and sodium storage. Nano Energy.

[135] Raccichini R., Varzi A., Passerini S., Scrosati B. (2015). The role of graphene for electrochemical energy storage. Nat. Mater..

[136] Liu Q., Sun J., Gao K., Chen N., Sun X., Ti D., Bai C., Cui R., Qu L. (2020). Graphene quantum dots for energy storage and conversion: From fabrication to applications. Mater. Chem. Front..

[137] Ovshinsky S.R., Fetcenko M.A., Ross J. (1993). A Nickel Metal Hydride Battery for Electric Vehicles. Science.

[138] Palacín M.R. (2018). Understanding ageing in Li-ion batteries: A chemical issue. Chem. Soc. Rev..

[139] Goodenough J.B., Park K.S. (2013). The Li-ion rechargeable battery: A perspective. J. Am. Chem. Soc..

[140] Kim J., Jang W., Kim J.H., Yang C.M. (2021). Synthesis of graphene quantum dots-coated hierarchical CuO microspheres composite for use as binder-free anode for lithium-ion batteries. Compos. Part B Eng..

[141] Wang F., Mao J. (2021). Extra Li-Ion Storage and Rapid Li-Ion Transfer of a Graphene Quantum Dot Tiling Hollow Porous SiO_2_ Anode. ACS Appl. Mater. Interfaces.

[142] Ansari S.A., Parveen N., Al-Othoum M.A.S., Ansari M.O. (2022). Inside–outside OH– incursion involved in the fabrication of hierarchical nanoflake assembled three-dimensional flower-like α-Co(OH)_2_ for use in high-performance aqueous symmetric supercapacitor applications. J. Adv. Res..

[143] Mousavi S.M., Hashemi S.A., Kalashgrani M.Y., Omidifar N., Bahrani S., Vijayakameswara Rao N., Babapoor A., Gholami A., Chiang W.-H. (2022). Bioactive Graphene Quantum Dots Based Polymer Composite for Biomedical Applications. Polymers.

[144] Ansari S.A., Parveen N., Al-Othoum M.A.S., Ansari M.O. (2022). Development of Binder Free Interconnected 3D Flower of NiZn2O4 as an Advanced Electrode Materials for Supercapacitor Applications. Crystals.

[145] Battistel A., Palagonia M.S., Brogioli D., la Mantia F., Trócoli R. (2020). Electrochemical Methods for Lithium Recovery: A Comprehensive and Critical Review. Adv. Mater..

[146] Peters J.F., Cruz A.P., Weil M. (2019). Exploring the economic potential of sodium-ion batteries. Batteries.

[147] Chao D., Zhu C., Xia X., Liu J., Zhang X., Wang J., Liang P., Lin J., Zhang H., Shen Z.X. (2015). Graphene quantum dots coated VO_2_ arrays for highly durable electrodes for Li and Na ion batteries. Nano Lett..

[148] Kong D., Wang Y., Huang S., von Lim Y., Zhang J., Sun L., Liu B., Chen T., Valdivia P., Alvarado Y. (2019). Surface modification of Na_2_Ti_3_O_7_ nanofibre arrays using N-doped graphene quantum dots as advanced anodes for sodium-ion batteries with ultra-stable and high-rate capability. J. Mater. Chem. A Mater..

[149] Deng G., Chao D., Guo Y., Chen Z., Wang H., Savilov S.v., Lin J., Shen Z.X. (2016). Graphene quantum dots-shielded Na_3_(VO)_2_(PO4)_2_F@C nanocuboids as robust cathode for Na-ion battery. Energy Storage Mater..

[150] Daryabari S., Mansouri S., Beheshtian J., Karimkhani M. (2021). A computational study on the novel defects of graphene quantum dot as a promising anode material for sodium ion battery. Mater. Chem. Phys..

[151] Liu W.W., Feng Y.Q., Yan X.b., Chen J.T., Xue Q.J. (2013). Superior micro-supercapacitors based on graphene quantum dots. Adv. Funct. Mater..

[152] Borenstein A., Hanna O., Attias R., Luski S., Brousse T., Aurbach D. (2017). Carbon-based composite materials for supercapacitor electrodes: A review. J. Mater. Chem. A Mater..

[153] Simonand P., Gogotsi Y. (2008). Materials for electrochemical capacitors. Nat. Mater..

[154] Muzaffar A., Ahamed M.B., Deshmukh K., Thirumalai J. (2019). A review on recent advances in hybrid supercapacitors: Design, fabrication and applications. Renew. Sustain. Energy Rev..

[155] Raza W., Ali F., Raza N., Luo Y., Kim K.H., Yang J., Kumar S., Mehmood A., Kwon E.E. (2018). Recent advancements in supercapacitor technology. Nano Energy.

[156] Poonam, Sharma K., Arora A., Tripathi S.K. (2019). Review of supercapacitors: Materials and devices. J. Energy Storage.

[157] Noori A., El-Kady M.F., Rahmanifar M.S., Kaner R.B., Mousavi M.F. (2019). Towards establishing standard performance metrics for batteries, supercapacitors and beyond. Chem. Soc. Rev..

[158] Pal A., Bhakat A., Chattopadhyay A. (2019). Zinc Ion-Induced Assembly of Crystalline Carbon Dots with Excellent Supercapacitor Performance. J. Phys. Chem. C.

[159] Masarapu C., Zeng H.F., Hung K.H., Wei B. (2009). Effect of temperature on the capacitance of carbon nanotube supercapacitors. ACS Nano.

[160] Gao W., Singh N., Song L., Liu Z., Reddy A.L.M., Ci L., Vajtai R., Zhang Q., Wei B., Ajayan P.M. (2011). Direct laser writing of micro-supercapacitors on hydrated graphite oxide films. Nat. Nanotechnol..

[161] Zhang S., Zhu J., Qing Y., Fan C., Wang L., Huang Y., Sheng R., Guo Y., Wang T., Pan Y. (2017). Construction of hierarchical porous carbon nanosheets from template-assisted assembly of coal-based graphene quantum dots for high performance supercapacitor electrodes. Mater. Today Energy.

[162] Ganganboina A.B., Chowdhury A.D., Doong R.A. (2017). New Avenue for Appendage of Graphene Quantum Dots on Halloysite Nanotubes as Anode Materials for High Performance Supercapacitors. ACS Sustain. Chem. Eng..

[163] Tian W., Zhu J., Dong Y., Zhao J., Li J., Guo N., Lin H., Zhang S., Jia D. (2020). Micelle-induced assembly of graphene quantum dots into conductive porous carbon for high rate supercapacitor electrodes at high mass loadings. Carbon.

[164] Lee K., Lee H., Shin Y., Yoon Y., Kim D., Lee H. (2016). Highly transparent and flexible supercapacitors using graphene-graphene quantum dots chelate. Nano Energy.

[165] Chen M., Wu B., Li D. (2020). Core–Shell Structured Cellulose Nanofibers/Graphene@Polypyrrole Microfibers for All-Solid-State Wearable Supercapacitors with Enhanced Electrochemical Performance. Macromol. Mater. Eng..

[166] Banerjee J., Dutta K., Kader M.A., Nayak S.K. (2019). An overview on the recent developments in polyaniline-based supercapacitors. Polym. Adv. Technol..

[167] Mondal S., Rana U., Malik S. (2015). Graphene quantum dot-doped polyaniline nanofiber as high performance supercapacitor electrode materials. Chem. Commun..

[168] Kumar R., Sahoo S., Joanni E., Singh R.K., Maegawa K., Tan W.K., Kawamura G., Kar K.K., Matsuda A. (2020). Heteroatom doped graphene engineering for energy storage and conversion. Mater. Today.

[169] Wang S., Shen J., Wang Q., Fan Y., Li L., Zhang K., Yang L., Zhang W., Wang X. (2019). High-Performance Layer-by-Layer Self-Assembly PANI/GQD-rGO/CFC Electrodes for a Flexible Solid-State Supercapacitor by a Facile Spraying Technique. ACS Appl. Energy Mater..

[170] Arthisree D., Madhuri W. (2020). Optically active polymer nanocomposite composed of polyaniline, polyacrylonitrile and green-synthesized graphene quantum dot for supercapacitor application. Int. J. Hydrogen Energy..

[171] Rahimpour K., Teimuri-Mofrad R. (2020). Novel hybrid supercapacitor based on ferrocenyl modified graphene quantum dot and polypyrrole nanocomposite. Electrochim. Acta.

[172] Abidin S.N.J.S.Z., Mamat S., Rasyid S.A., Zainal Z., Sulaiman Y. (2018). Fabrication of poly(vinyl alcohol)-graphene quantum dots coated with poly(3,4-ethylenedioxythiophene) for supercapacitor. J. Polym. Sci. A Polym. Chem..

[173] Abidin S.N.J.S.Z., Mamat M.S., Rasyid S.A., Zainal Z., Sulaiman Y. (2018). Electropolymerization of poly(3,4-ethylenedioxythiophene) onto polyvinyl alcohol-graphene quantum dot-cobalt oxide nanofiber composite for high-performance supercapacitor. Electrochim. Acta..

[174] Jia H., Cai Y., Lin J., Liang H., Qi J., Cao J., Feng J., Fei W.D. (2018). Heterostructural Graphene Quantum Dot/MnO_2_ Nanosheets toward High-Potential Window Electrodes for High-Performance Supercapacitors. Adv. Sci..

[175] Zhang W., Yang Y., Xia R., Li Y., Zhao J., Lin L., Cao J., Wang Q., Liu Y., Guo H. (2020). Graphene-quantum-dots-induced MnO_2_ with needle-like nanostructure grown on carbonized wood as advanced electrode for supercapacitors. Carbon.

[176] Ganganboina A.B., Park E.Y., Doong R.A. (2020). Boosting the energy storage performance of V_2_O_5_ nanosheets by intercalating conductive graphene quantum dots. Nanoscale.

[177] Ruiyi L., Keyang H., Yongqiang Y., Haiyan Z., Zaijun L. (2021). Atomically dispersed RuO2-tryptophan functionalized graphene quantum dot-graphene hybrid with double Schottky heterojunctions for high performance flexible supercapacitors. Chem. Eng. J..

[178] Ganganboina A.B., Chowdhury A.D., Doong R.A. (2017). Nano assembly of N-doped graphene quantum dots anchored Fe_3_O_4_/halloysite nanotubes for high performance supercapacitor. Electrochim. Acta.

[179] Moghimian S., Sangpour P. (2020). One-step hydrothermal synthesis of GQDs-MoS_2_ nanocomposite with enhanced supercapacitive performance. J. Appl. Electrochem..

[180] Regulska E., Breczko J., Basa A., Szydlowska B., Kakareko K., Rydzewska-Rosołowska A., Hryszko T. (2022). Graphene-quantum-dots-decorated NiAl_2_O_4_ nanostructure as supercapacitor and electrocatalyst in biosensing. Mater. Today Commun..

[181] Ashourdan M., Semnani A., Hasanpour F., Moosavifard S.E. (2021). Synthesis of CuMnO_2_/graphene quantum dot nanocomposites as novel electrode materials for high performance supercapacitors. J. Energy Storage.

[182] Zhang M., Liu W., Liang R., Tjandra R., Yu A. (2019). Graphene quantum dot induced tunable growth of nanostructured MnCo_2_O_4.5_ composites for high-performance supercapacitors. Sustain. Energy Fuels.

[183] Wang H., Yang Y., Zhou X., Li R., Li Z. (2017). NiCo_2_S_4_/tryptophan-functionalized graphene quantum dot nanohybrids for high-performance supercapacitors. New J. Chem..

[184] Sangabathula O., Sharma C.S. (2020). One-pot hydrothermal synthesis of molybdenum nickel sulfide with graphene quantum dots as a novel conductive additive for enhanced supercapacitive performance. Mater. Adv..

[185] Zhang S., Li Y., Song H., Chen X., Zhou J., Hong S., Huang M. (2016). Graphene quantum dots as the electrolyte for solid state supercapacitors. Sci. Rep..

